# Efficient Photocatalytic Hydrogen Evolution Enabled by Defect‐ and Interface‐Induced Dual Built‐in Electric Fields in a ZnIn_2_S_4_/1T‐2H WS_2_ Heterojunction

**DOI:** 10.1002/smll.202514954

**Published:** 2026-03-23

**Authors:** Ning Li, Jinyu Zhang, Jiafeng Ma, Chaorui Xue, Qing Chang, Lei Liu, Xiangqian Fan, Caihong Hao, Shaobin Wang, Shengliang Hu, Wenjie Tian

**Affiliations:** ^1^ School of Energy and Power Engineering & State Key Laboratory of Coal and CBM Co‐Mining North University of China Taiyuan China; ^2^ School of Chemical Engineering Adelaide University Adelaide South Australia Australia

**Keywords:** defect engineering, dual built‐in electric fields, p‐n homojunction, type‐II heterojunction, ZnIn_2_S_4_/1T‐2H WS_2_ composite

## Abstract

Efficient photocatalysis requires coordinated regulation of charge transport across both bulk and interfacial regions. Here, we introduce an in situ hydrothermal‐solvothermal method that simultaneously creates a defect‐induced p‐n homojunction in ZnIn_2_S_4_ (ZIS) and a type‐II heterojunction with hybrid 1T‐2H WS_2_, forming antiparallel internal‐interfacial built‐in electric fields (BIEF) that are confirmed by spectroscopic, electronic, optical, and theoretical analyses. Under the guidance of this dual‐field coupling effect, photoexcited carriers undergo more efficient separation, enabling the effective modulation of bulk carrier migration and directing electrons toward sulfur‐vacancy (S_v_) sites in ZIS for efficient hydrogen evolution. The hybrid 1T‐2H phases of WS_2_ further enhance light absorption and facilitate rapid charge generation and transfer, reinforcing the dual‐BIEF‐driven transport pathway. The optimized ZIS/WS_2_ photocatalyst achieves a hydrogen evolution rate of 44.97 mmol g^−1 ^h^−1^ and an apparent quantum efficiency of 24.54% at 400 nm. This work establishes antiparallel dual‐BIEF engineering combined with 1T‐2H hybrid‐phase modulation as a platform for directional charge‐dynamic control, offering a pathway toward efficient solar‐to‐hydrogen conversion.

## Graphical abstract

An N, S_v_‐ZnIn_2_S_4_/1T‐2H WS_2_ composite photocatalyst is constructed via an in situ hydrothermal‐solvothermal strategy, simultaneously forming a defect‐induced p‐n homojunction in ZnIn_2_S_4_ and a type‐II heterojunction with hybrid 1T/2H WS_2_. The coupled dual built‐in electric fields promote charge separation and direct photoexcited electrons toward sulfur‐vacancy sites in ZnIn_2_S_4_, leading to enhanced photocatalytic hydrogen evolution.

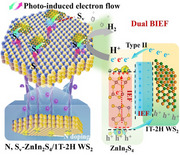

## 1 Introduction

The efficient utilization of hydrogen energy is one of the core strategies for addressing the global energy crisis and environmental issues [1, 2]. However, developing low‐cost and sustainable hydrogen production technologies remains a major technical bottleneck for large‐scale green hydrogen deployment. Among various approaches, solar‐driven catalytic water splitting has risen as a highly attractive renewable route for green hydrogen generation [3, 4]. Unfortunately, the photocatalytic activity is severely limited by the insufficient charge separation and random carrier transfer [5]. An ideal photocatalytic system should efficiently separate photogenerated carriers and direct them precisely to active sites, which remains a long‐standing, pivotal challenge in this field.

Among various semiconductor photocatalysts, ZnIn_2_S_4_ (abbreviated as ZIS) has garnered extensive research attention owing to its excellent photostability, well‐matched band structure, and prominent reduction capability [6, 7, 8, 9, 10, 11]. Yet, the sluggish charge carrier separation and migration kinetics of ZIS lead to unsatisfactory photocatalytic performance. Defect engineering and elemental doping are commonly used to tailor the electronic structure of ZIS [6, 7, 8, 9, 11, 12, 13, 14, 15, 16, 17]. For instance, sulfur vacancies (S_v_) can improve light absorption and introduce electron‐trapping states [5, 18], whereas N dopant incorporation forms hole‐trapping centers that effectively inhibit charge carrier recombination [15]. Although these strategies improve local carrier separation, they do not establish a continuous driving force for directional charge transport. Consequently, photogenerated carriers in defect‐engineered ZIS still migrate in a largely diffusive manner, and excessive defects may even act as recombination centers, thereby severely limiting the extent of photocatalytic performance improvement. From a transport perspective, built‐in electric fields (BIEFs) are essential to enable directional separation and migration of charge carriers. A BIEF can be generated either internally within a semiconductor or at the interface between two semiconductors with different work functions [10]. An internal BIEF, formed by a p‐n homojunction, induces continuous band bending across the bulk and provides a long‐range driving force for carrier migration [14, 19]. Even so, a standalone internal BIEF is often inadequate for efficient extraction of charge carriers from inside to surface catalytic sites. Therefore, simultaneously integrating internal and interfacial BIEFs into a single system is required to achieve continuous and efficient charge separation and transport from the bulk to the surface.

In this context, S_v_ and N dopants have been reported to induce *n*‐type and *p*‐type characteristics, respectively, making the construction of an internal p‐n homojunction feasible for internal BIEF [13, 14, 15]. Meanwhile, constructing heterojunctions between ZIS and semiconductors with well‐matched energy levels, like transition metal chalcogenides (MoSe_2_, MoS_2_, SnS_2_) [10, 11, 13, 16], has proven to be an effective approach for interficial BIEF. Among them, WS_2_ is a particularly promising cocatalyst or semiconductor to couple with ZIS due to its layered structure, good light absorption, and adjustable band structure (band gap ∼1.4–2.0 eV) [20, 21]. Notably, WS_2_ can be engineered into mixed metallic 1T and semiconducting 2H phases, which endow it with better electrical conductivity, higher carrier mobility, and broader light absorption than single‐phase WS_2_ [21, 22]. However, ZIS coupled with phase‐hybrid WS_2_ (1T‐2H) remains an unaddressed research topic to date, and how such phase‐hybrid heterostructures regulate band alignment, BIEFs, and charge‐transfer pathways in ZIS‐based systems remains unclear.

Herein, an N, S_v_‐ZIS/1T‐2H WS_2_ (abbreviated as N, S_v_‐ZIS/WS_2_) photocatalyst was constructed to integrate an internal p‐n homojunction and an interficial type‐II heterojunction through a combined regulation of N doping, S_v_ engineering, and ZIS/1T‐2H WS_2_ coupling. Our findings revealed that the resulting antiparallel dual‐BIEFs enable efficient separation of photogenerated electron‐hole pairs and direct photoexcited electrons toward the S_v_ sites in ZIS. The photocatalytic hydrogen evolution mechanism was systematically elucidated through combined experimental analyses and density functional theory (DFT) calculations. The optimized N, S_v_‐ZIS/WS_2_ delivers a hydrogen evolution rate of 44.97 mmol g^−1 ^h^−1^, which is 8.53‐fold higher than that of pristine ZIS, along with an apparent quantum efficiency (AQE) of 24.54% under 400 nm monochromatic light irradiation.

## 2 Results and Discussion

### 2.1 Preparation and Characterization of the Photocatalysts

**FIGURE 1:**
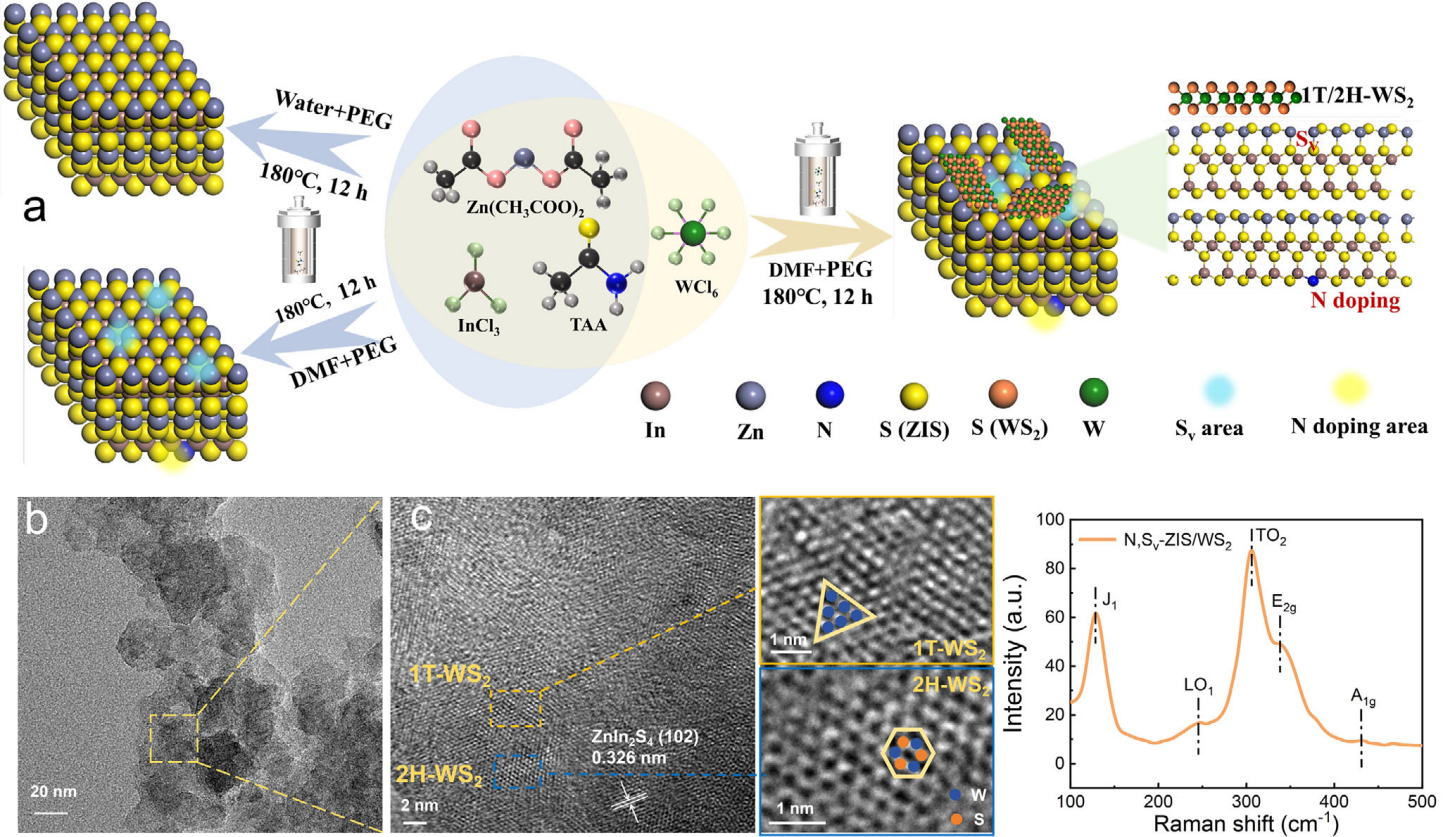
(a) Schematic diagram of pristine ZIS, N, S_v_‐ZIS, and N, S_v_‐ZIS/WS_2_ heterojunction preparation. (b) TEM, (c) high‐resolution TEM (HR‐TEM). (d) Raman spectrum of N, S_v_‐ZIS/WS_2_.

Figure 1a illustrates the schematic design of the catalysts. Pristine ZIS, N, S_v_‐ZIS, and N, S_v_‐ZIS/WS_2_ samples were fabricated via a facile one‐step *in situ* hydrothermal‐solvothermal method. First, pristine ZIS was synthesized in an aqueous solvent. Thioacetamide (TAA) and N, N‐dimethylformamide (DMF) decomposed to generate H_2_S and (CH_3_)_2_NH under high temperature and pressure conditions of the hydrothermal reaction. Subsequently, H_2_S reacted with (CH_3_)_2_NH to form [(CH_3_)_2_NH_2_]^+^[HS]^−^. Owing to the strong reducibility of HS^−^ and the N doping effect of DMF, S vacancies and N dopants were simultaneously introduced into the ZIS lattice [14, 23]. Specifically, due to their unsaturated bonds and high reactivity, sulfur vacancies act as precise nucleation sites for WS_2_ during the hydrothermal reaction, thereby promoting the subsequent reaction between WCl_6_ and TAA to form WS_2_, and ultimately yielding the N, S_v_‐ZIS/WS_2_ sample.

As illustrated in the scanning electron microscopy (SEM) images of Figure S1a, the synthesized pristine ZIS exhibits a nanoflower‐like structure assembled from interlaced nanosheets. In contrast, the N, S_v_‐ZIS prepared in DMF solvent displays a bulk‐like structure with a rough surface (Figure S1b). This morphological difference may arise from the following mechanism. The carbonyl group (C = O) in DMF and the formed (CH_3_)_2_NH can form stable complexes with Zn^2^^+^ ions, which slows down the polycondensation rate of Zn^2^^+^ and delays the nucleation process. Slower reaction kinetics favor particle aggregation over nucleation, leading to the formation of a blocky structure. After combining with WS_2_, the SEM image reveals that the surface of N, S_v_‐ZIS/WS_2_ exhibits a densely packed and layered sheet‐like structure (Figure S1c), leading to the exposure of more surface active sites. The specific surface areas and pore size distributions of N, S_v_‐ZIS, and N, S_v_‐ZIS/WS_2_ were characterized using N_2_ adsorption–desorption measurements. As shown in Figure S2 and Table S1, all samples exhibit typical type‐IV isotherms with H3 hysteresis loops, suggesting mesoporous structures. N, S_v_‐ZIS/WS_2_ possesses a slightly higher specific surface area (SSA) than N, S_v_‐ZIS, and higher SSA tends to facilitate active site exposure and photocatalytic hydrogen evolution performance [24].

Figure S3 illustrates the energy‐dispersive X‐ray spectroscopy (EDS) elemental mapping of the N, S_v_‐ZIS/WS_2_ sample. The uniform distribution of Zn, In, S, and N elements is evident, while the spatial distribution of W and S elements confirms the successful loading of WS_2_ onto the ZIS. To further elucidate the microstructure of the N, S_v_‐ZIS/WS_2_ samples, HR‐TEM was employed for detailed characterization. As shown in Figure 1b, c, lattice fringes from distinct regions of the N, S_v_‐ZIS/WS_2_ sample are clearly resolved. A lattice spacing of 0.326 nm corresponds to the (102) crystallographic plane of ZIS [13]. Furthermore, the coexistence of octahedral 1T‐phase WS_2_ (triangular lattice regions) and trigonal prismatic 2H‐phase WS_2_ (honeycomb lattice regions) was confirmed by the zoom‐in HRTEM images (Figure 1c) [25]. To obtain crystallographic information of the samples, XRD analysis was performed on the synthesized photocatalysts. X‐ray diffraction (XRD) patterns of ZIS, N, S_v_‐ZIS, and N, S_v_‐ZIS/WS_2_ are displayed in Figure S4. All characteristic diffraction peaks of ZIS and N, S_v_‐ZIS exhibit perfect alignment with the standard card of ZIS (JCPDS: 65–2023) [13, 26]. For the N, S_v_‐ZIS/WS_2_ sample, in addition to the characteristic ZIS peaks, distinct WS_2_ diffraction peaks are observed in the XRD pattern, which conclusively verifies the successful integration of both materials. Notably, the diffraction peaks at 2θ = 14.3°, 32.7°, 43.9°, and 58.4° are attributed to the (002), (100), (006) and (110) crystal planes of 2H‐phase WS_2_, respectively [21], while the characteristic peak at 2θ = 31.8° corresponds to the (006) plane of 1T‐WS_2_ [25]. Figure 1d depicts the Raman spectrum of the synthesized N, S_v_‐ZIS/WS_2_ heterostructure. The characteristic peaks at 242 and 300 cm^−1^ correspond to the longitudinal optical (LO_1_) and transverse optical (TO_2_) modes of ZIS, respectively [27]. The strong peaks at 335 and 430 cm^−1^ are attributed to the E_2g_ (in‐plane) and A_1g_ (out‐of‐plane) vibrational modes of 2H‐WS_2_ [25]. Notably, the Raman peak at 125 cm^−1^ originates from the J_1_ mode of 1T‐WS_2_ [22]. This result is consistent with the transmission electron microscopy (TEM) and XRD analysis, further confirming the coexistence of 1T‐2H phases in WS_2_ and the successful integration of WS_2_ with ZIS.

### 2.2 Composition and Chemical State Analysis

**FIGURE 2:**
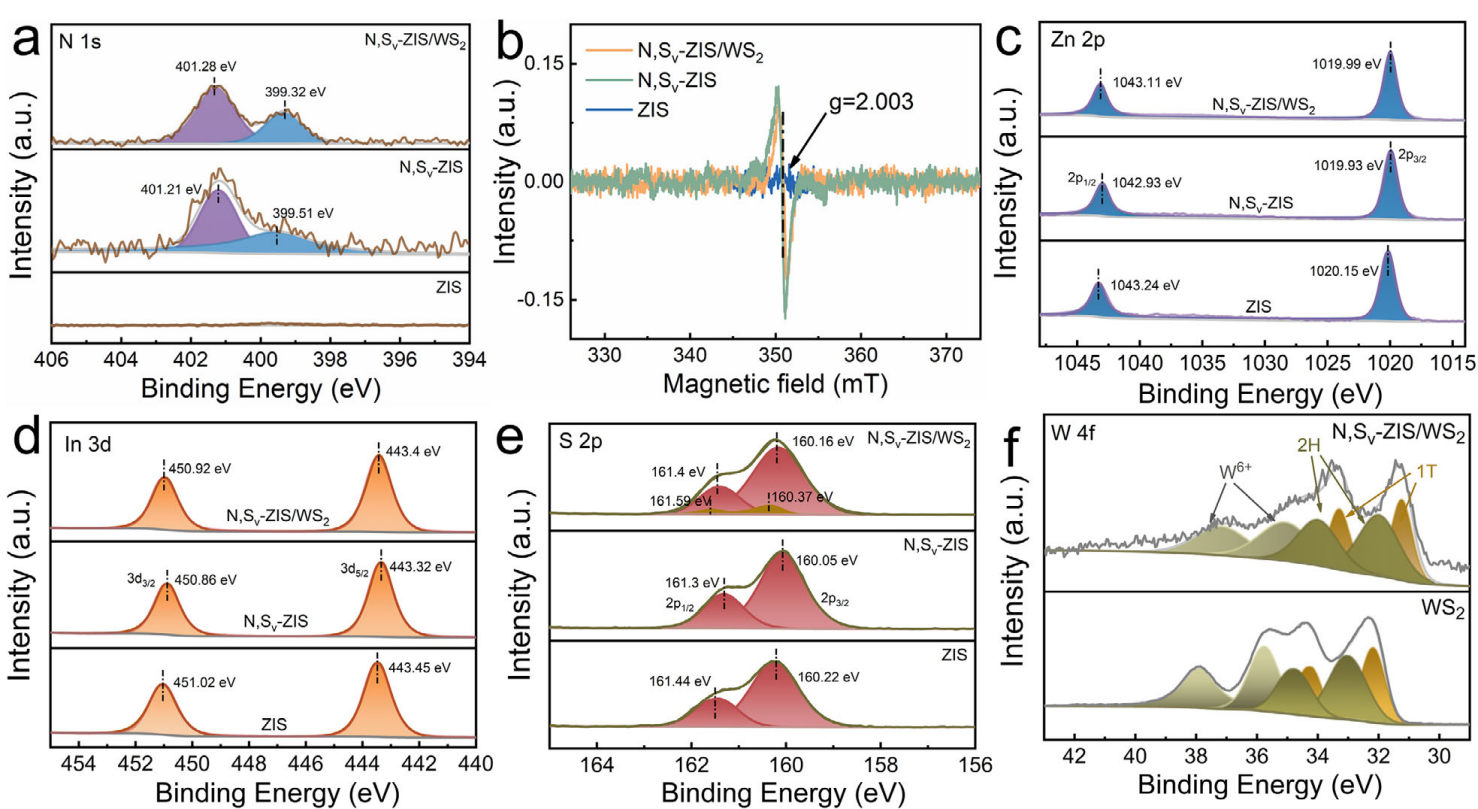
(a) N 1s XPS spectra of ZIS, N, S_v_‐ZIS, and N, S_v_‐ZIS/WS_2_ samples. (b) EPR spectra of ZIS, N, S_v_‐ZIS, and N, S_v_‐ZIS/WS_2_ samples. (c‐f) Zn 2p, In 3d, S 2p, and W 4f XPS spectra of ZIS, N, S_v_‐ZIS, and N, S_v_‐ZIS/WS_2_ samples.

The elemental composition and chemical states were analyzed by using X‐ray photoelectron spectroscopy (XPS). As shown in the XPS survey spectrum of Figure S5, peaks corresponding to Zn, In, and S elements are observed in the ZIS, N, S_v_‐ZIS, and N, S_v_‐ZIS/WS_2_ samples, while distinct N peaks are found in the latter two samples. As shown in Figures 2a, N 1s with deconvoluted peaks at 399.5 and 401.2 eV can be attributed to metal‐N bonds and the presence of N species, respectively [14, 15]. This verified the successful incorporation of N dopants into N, S_v_‐ZIS, and N, S_v_‐ZIS/WS_2_. Meanwhile, according to the XPS quantitative elemental analysis summarized in Table S2, the atomic ratios of Zn, In, and S are 1:2.01:4 for pristine ZIS and 1:2.01:3.79 for N, S_v_‐ZIS, suggesting the formation of S_v_. To further confirm the presence of S_v_ in the N, S_v_‐ZIS, and N, S_v_‐ZIS/WS_2_ samples, electron paramagnetic resonance (EPR) tests were conducted. As shown in Figure 2b, almost no significant EPR signal intensity is observed in the pristine ZIS. Notably, the N, S_v_‐ZIS and N, S_v_‐ZIS/WS_2_ samples exhibit the markedly enhanced EPR signals at a g‐factor of 2.003, confirming the appearance of S vacancies [13, 28]. Notably, N, S_v_‐ZIS/WS_2_ exhibits a slight decrease in the intensity compared with N, S_v_‐ZIS, indicating that S_v_ sites induce the deposition of WS_2_ at the vacancy locations, thereby reducing the number of unpaired electrons [16]. Figure 2c,d illustrate the high‐resolution XPS spectra of Zn 2p and In 3d. The binding energies at 1043.11 and 1019.99 eV in the Zn 2p spectrum correspond to Zn 2p_1/2_ and Zn 2p_3/2_ of N, S_v_‐ZIS/WS_2_ sample. In 3d spectrum shows two peaks at 450.92 and 443.3 eV, which are attributed to In 3d_3/2_ and In 3d_5/2_, respectively [29]. Similarly, the S 2p spectrum of N, S_v_‐ZIS/WS_2_ can be deconvoluted into two spin–orbit doublets with binding energies of 160.16 eV (160.37 eV) and 161.4 eV (161.59 eV), attributed to the S 2p_3/2_ and S 2p_1/2_ components of ZIS and WS_2_ in the heterostructure (Figure 2e).

Notably, compared to pristine ZIS, both N, S_v_‐ZIS and N, S_v_‐ZIS/WS_2_ samples exhibit significant negative shifts in their S 2p_3/2_ and S 2p_1/2_ binding energies, confirming the generation of S_v_. These S_v_ species can act as strong electron‐donating centers, facilitating the transfer of electrons from the S_v_ sites to the ZIS lattice [30]. This process increases the electron cloud density around sulfur atoms in ZIS [30], consequently leading to the observed decrease in binding energy of S 2p. This is consistent with DFT calculation results in Figure 6a. In addition, the W 4f spectrum of N, S_v_‐ZIS/WS_2_ (Figure 2f) was resolved into three spin–orbit doublets. The peaks located at 32.18 and 34.23 eV are assigned to W 4f_7/2_ and W 4f_5/2_ of the 1T WS_2_, while the peaks at 33.02 and 34.75 eV correspond to W 4f_7/2_ and W 4f_5/2_ of the 2H WS_2_ [22, 25]. After calculation, the proportion of the 2H‐phase accounts for approximately 55.65%, indicating that the WS_2_ consists of a mixed‐phase structure (1T and 2H). Compared to WS_2_, the W 4f peaks in the N, S_v_‐ZIS/WS_2_ shift toward lower binding energies. In contrast, the binding energies of Zn 2p, In 3d, and S 2p belonging to N, S_v_‐ZIS/WS_2_ increase in comparison to those of N, S_v_‐ZIS (Figure 2c–e). The binding energy shift characteristics demonstrate that interfacial interaction occurs at the WS_2_‐ZIS interface in the N, S_v_‐ZIS/WS_2_ heterojunction, with electrons spontaneously migrating from ZIS to WS_2_ due to the differences in Fermi energy levels (*E*_f_) and surface charge density [10]. This charge transfer results in the band bending and forms a built‐in electric field at their interface [31].

### 2.3 Photocatalytic H_2_ Evolution Performance

**FIGURE 3:**
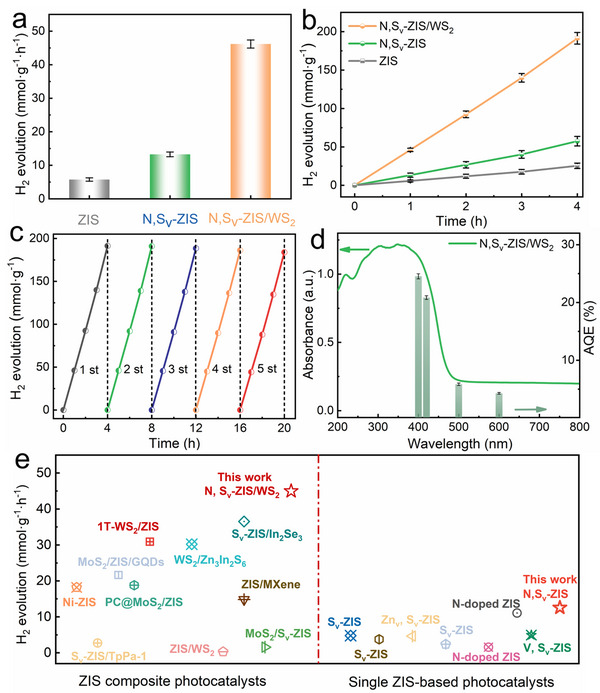
(a, b) H_2_ evolution rates and H_2_ evolution over time of different samples. (c) Recycling tests of photocatalytic H_2_ evolution of N, S_v_‐ZIS/WS_2_. (d) wavelength‐dependent AQE at the wavelength of 400, 420, 500, and 600 nm over N, S_v_‐ZIS/WS_2_ sample. (e) Comparison of H_2_ evolution rates with previously reported ZIS‐based photocatalysts, as summarized in Table S5.

The photocatalytic H_2_ production tests were conducted under simulated solar light irradiation (AM 1.5, 100 mW/cm^2^). Before the performance test, we first optimized the concentrations of N doping and S_v_ by adjusting the volume ratio of water to DMF (dual source for N doping and S_v_). Results show that the photocatalytic performance of N, S_v_‐ZIS sample synthesized in 10 mL DMF is optimal (Figure S6). On this basis, we further regulated the amount of WCl_6_ (the precursor of WS_2_) to tailor the composite ratio of ZIS/WS_2_. The catalytic performance of the composites is shown in Figure S7. Specifically, the addition of 64 mg of WCl_6_ was identified as the optimal ZIS/WS_2_ composite ratio for N, S_v_‐ZIS/WS_2_ heterostructure. As shown in Figure 3a, pure ZIS exhibits poor hydrogen evolution activity due to severe carrier recombination, reaching only 5.27 mmol·g^−1^·h^−1^. After introducing S_v_ and N dopants, the photocatalytic H_2_ evolution rate of N, S_v_‐ZIS sample is increased to 12.57 mmol·g^−1^·h^−1^, which fully confirms the positive effect of N doping and S_v_ on promoting the catalytic activity of ZIS. Notably, the N, S_v_‐ZIS/WS_2_ heterostructure with the above optimal composite ratio achieves an H_2_ production rate of 44.97 mmol·g^−1^·h^−1^ and a total H_2_ production amount of 191.33 mmol·g^−1^ within 4 h (Figure 3a,b), which is about 8.55 and 7.53 times higher than those of pristine ZIS, respectively. In addition, the optimal N, S_v_‐ZIS/WS_2_ also demonstrated excellent photocatalytic stability during five consecutive H_2_ production catalytic cycles (Figure 3c).

Based on the XRD and XPS characterization results of the N, S_v_‐ZIS/WS_2_ sample after photocatalytic testing, the structural and compositional features remain consistent with its pre‐reaction state (Figure S8). Meanwhile, N, S_v_‐ZIS/WS_2_ sample keeps the packed and layered sheet‐like morphology, and the 1T‐WS_2_ (triangular lattice regions) and 2H‐WS_2_ (honeycomb lattice regions) phases were also observed in the HR‐TEM images after reaction (Figures S8 and S9). These results verify the excellent stability of N, S_v_‐ZIS/WS_2_ catalyst. Subsequently, the apparent quantum efficiency (AQE) of different samples was measured and calculated at specific monochromatic light wavelengths (400, 420, 500, and 600 nm, Table S3). As displayed in Figure 3d and Figure S10, the AQE values exhibit a strong correlation with the absorption spectrum of the samples (absorbance values in 400–700 nm are provided in Table S4‐1, ‐2). Notably, the AQE values of N, S_v_‐ZIS/WS_2_ are 24.54% at 400 nm and 20.85% at 420 nm, much higher than N, S_v_‐ZIS. Owing to the weak tail absorption of N, S_v_‐ZIS/WS_2_ and N, S_v_‐ZIS, the AQE values of these samples are calculated as 4.28% and 1.52% at 600 nm, respectively. Comparative analysis shows that this N, S_v_‐ZIS/WS_2_ heterostructure exhibits superior photocatalytic hydrogen evolution rates over numerous other ZIS‐based catalysts (Figure 3e) [8, 11, 13, 14, 15, 16, 18, 22, 23, 26, 32, 33, 34, 35, 36, 37]. The key experimental parameters involved in these comparisons, including light source, catalyst loading, reactor volume, and type/concentration of sacrificial agent, are systematically summarized in Table S5. This result underscores the significant potential of the N, S_v_‐ZIS/WS_2_ heterostructure for photocatalytic applications.

### 2.4 The Intrinsic Origin of the Enhanced Catalytic Activity

**FIGURE 4:**
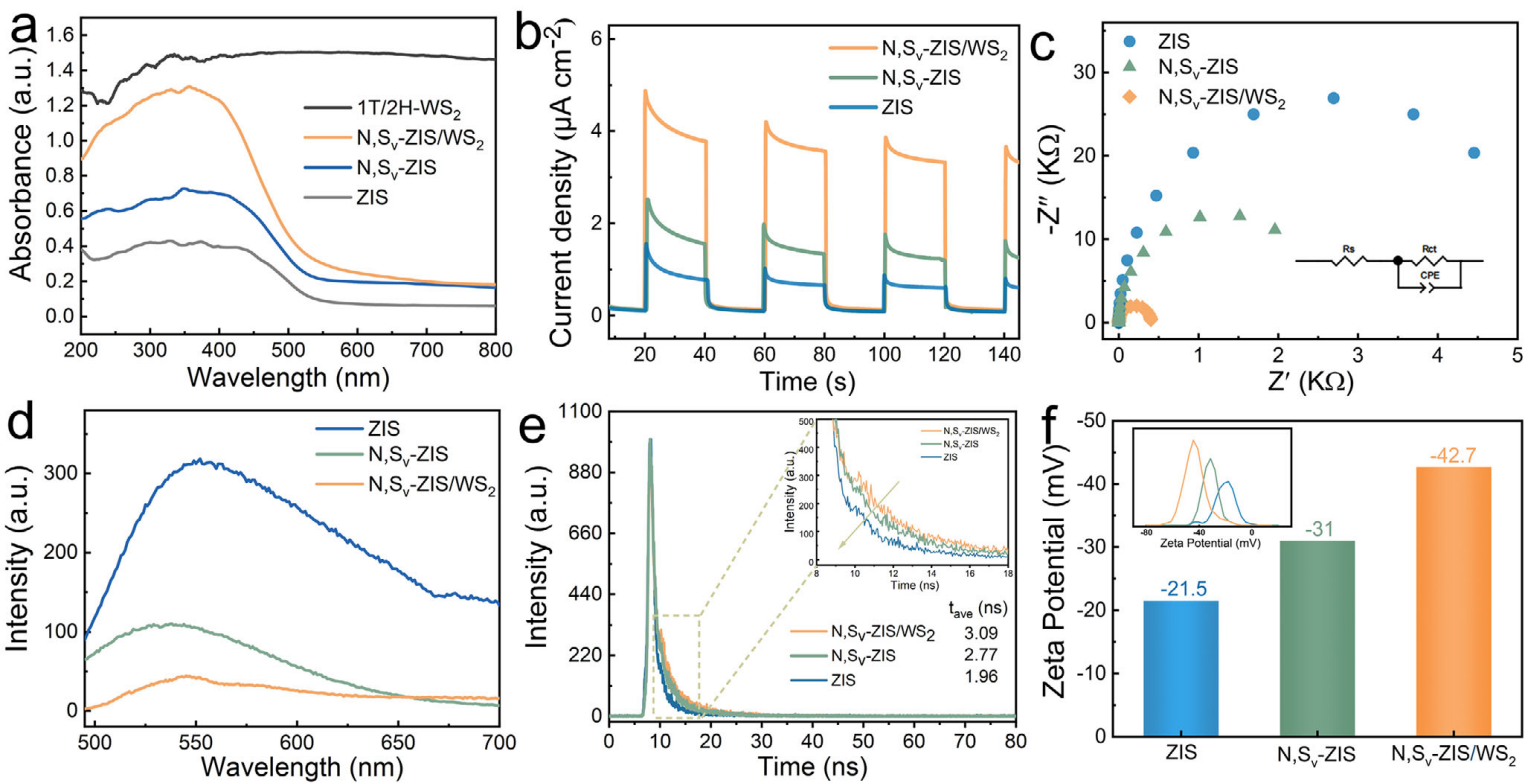
(a) UV–vis absorption spectra. (b) Photocurrent response and (c) EIS plots of different samples, respectively. (d) PL and (e) TRPL of ZIS, N, S_v_‐ZIS, and N, S_v_‐ZIS/WS_2_ samples. (f) Zeta potential of different samples (The inset figure is the testing curves).

The intrinsic reasons for the enhanced photocatalytic hydrogen evolution performance of the N, S_v_‐ZIS/WS_2_ heterostructure were elucidated through a series of characterizations. Figure 4a depicts the UV–vis absorption spectra of ZIS, N, S_v_‐ZIS, and N, S_v_‐ZIS/WS_2_. It is evident that ZIS primarily absorbs ultraviolet light, while N, S_v_‐ZIS exhibits enhanced light absorption compared to ZIS. This indicates that the introduction of S_v_ and N dopants can effectively modulate the optical properties of ZIS. 1T‐2H WS_2_ demonstrates strong absorption across the entire UV–vis range, which is consistent with the reported literature [22]. Furthermore, after combining with WS_2_, the composite N, S_v_‐ZIS/WS_2_ shows significantly enhanced light absorption ability in contrast to N, S_v_‐ZIS. This enhanced light absorption is more conducive to the generation of photogenerated carriers, which helps improve the photocatalytic hydrogen evolution performance [10, 38]. Figure 4b presents the photocurrent responses of these samples under intermittent light irradiation. It can be observed that the photocurrent density of N, S_v_‐ZIS/WS_2_ is higher than that of N, S_v_‐ZIS and ZIS samples, which implies more efficient photoinduced carrier separation and migration [11, 34]. The charge transfer property was further analyzed by using electrochemical impedance spectroscopy (EIS). As shown in Figure 4c, the arc radius of N, S_v_‐ZIS/WS_2_ is significantly smaller than those of ZIS and N, S_v_‐ZIS, indicating the effective reduction of the interfacial charge transfer resistance after the loading of 1T‐2H WS_2_, which facilitates the accelerated transfer of photogenerated charge carriers [10, 33].

To investigate the separation efficiency of photogenerated charge carriers in ZIS, N, S_v_‐ZIS, and N, S_v_‐ZIS/WS_2_ photocatalysts, photoluminescence (PL) spectroscopy characterization was performed. The PL spectra show that pristine ZIS exhibits significantly higher PL intensity under 375 nm excitation wavelength (Figure 4d), suggesting a high recombination rate of photogenerated charge carriers. Compared with ZIS, the emission peak intensity of N, S_v_‐ZIS decreases remarkably, which is attributed to the p‐n homojunction BIEF and the trap effects of S_v_ and N defective sites promoting the separation of photogenerated charge carriers. Notably, after loading 1T‐2H WS_2_ onto ZIS, the formation of interfacial BIEF further enhances the photogenerated charge separation efficiency. Meanwhile, the excellent conductivity of 1T‐WS_2_ enables faster carrier transfer in the N, S_v_‐ZIS/WS_2_ heterostructure [22, 39]. Consequently, N, S_v_‐ZIS/WS_2_ exhibits the lowest PL intensity. For the acquisition of deeper insight into the charge dynamics, the time‐resolved photoluminescence (TRPL) spectra measurements were conducted (Figure 4e). The average PL lifetime (*τ*_a_) was calculated according to the following equation 1 [34]:

(1) $$\tau_{a}=\frac{A_{1}\tau_{1}^{2}+A_{2}\tau_{2}^{2}}{A_{1}\tau_{1}+A_{2}\tau_{2}}\;$$

where *A*_1_ and *A*_2_ represent the corresponding amplitudes and τ_1_ and *τ*_2_ represent the short and long‐lived lifetimes, respectively. The fitting parameters (*χ* ^2^) for all samples are close to 1. The average PL lifetime of N, S_v_‐ZIS/WS_2_ (3.09 ns) is longer than that of pristine ZIS (1.96 ns) and N, S_v_‐ZIS (2.77 ns), demonstrating there existed the effective separation of photoinduced carriers in N, S_v_‐ZIS/WS_2_ through dual BIEFs [40], which inhibits the charge recombination and improves the photocatalytic efficiency [6, 8, 12, 34]. Moreover, considering the direct correlation between the adsorption of surface hydrogen ions (H^+^) and photocatalytic hydrogen evolution performance, we employed Zeta potential characterization to measure the surface electronegativity of different samples. As illustrated in Figure 4f, Zeta potentials of ZIS, N, S_v_‐ZIS, and N, S_v_‐ZIS/WS_2_ are −21.5, −31, and −42.7 mV, respectively. Notably, N, S_v_‐ZIS/WS_2_ exhibited a significantly more negative potential, followed by N, S_v_‐ZIS, and ZIS in descending order. This enhanced surface electronegativity facilitates the adsorption of positively charged H^+^ ions, thereby promoting the photocatalytic water splitting process for hydrogen production [14].

### 2.5 Insights Into the Antiparallel Internal‐Interfacial Dual Built‐in Electric Fields

**FIGURE 5:**
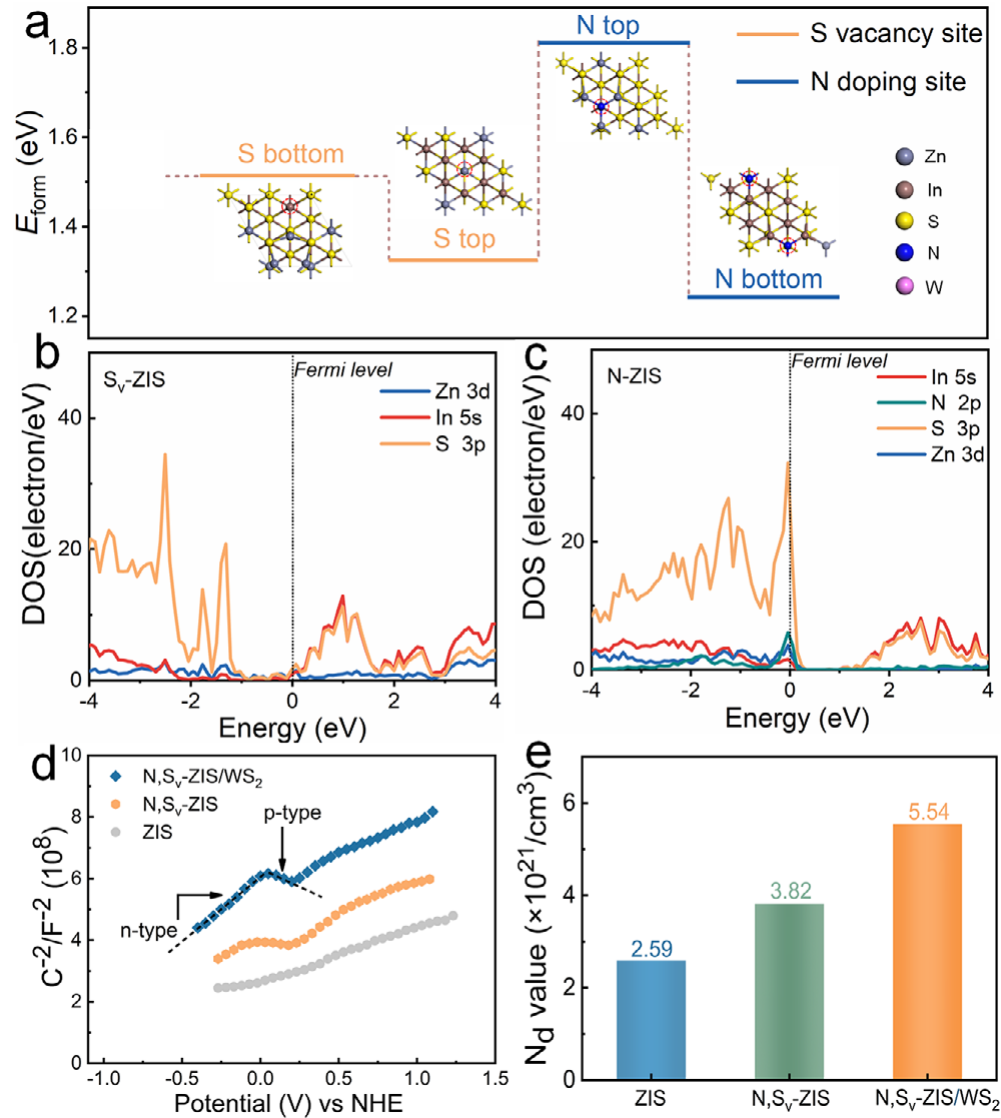
(a) *E*_form_ values of S_v_ and N dopant introduced into the ZIS framework at different positions. (b, c) Partial density of states (PDOS) of S_v_‐ZIS and N‐ZIS, respectively. (d, e) MS plots and the calculated *N*_d_ values of ZIS, N, S_v_‐ZIS, and N, S_v_‐ZIS/WS_2_ samples.

Through an integrated strategy combining theoretical calculations and experimental investigations, we systematically elucidate the synergistic effects of S vacancies, N doping, interfacial interactions, and charge transfer mechanisms in N, S_v_‐ZIS/WS_2_. First, DFT simulations were conducted to elucidate the spatial localization and functional implications of S_v_ and N dopants within the N, S_v_‐ZIS atomic framework. Comparative analysis of formation energies (*E*_form_) for vacancy generation and dopant substitution revealed site‐specific preferences (Figure 5a). The Zn‐coordinated sulfur atoms at the top layer exhibit lower *E*_form_, indicating their preferential escape tendency during synthesis. Conversely, the smaller *E*_form_ of incoordinated sulfur sites at the bottom layer demonstrates a higher propensity for N incorporation at this position. Figure 5b,c show the calculated partial density of states (PDOS) for S_v_‐engineered ZIS and N‐doped ZIS. Optimal incorporation of S vacancy into ZIS structure introduces donor states near the Fermi level, indicating the n‐type behavior [41]. Moreover, the acceptor level originated predominantly from In 5s, S 3p, Zn 3d, and N 2p hybrid orbitals that pass through the Fermi level, making the Fermi level shift down toward the valence band region, endowing a p‐type behavior [14, 15]. Therefore, it can be deduced that the configuration of p‐n charge properties is realized in the ZIS via N doping and S_v_ modification. The charge conductivity nature was further ascertained by Mott–Schottky (MS) analysis.

As shown in Figure 5d, the positive slope of the MS curve for ZIS reflects its characteristic as an n‐type semiconductor. By comparison, N, S_v_‐ZIS exhibits an inverted “V”‐shaped curve, which arises from the presence of S_v_ and N dopants, which is a characteristic of p‐n charge behavior, indicating the formation of an internal p‐n homojunction in N, S_v_‐ZIS [14]. Notably, a similar inverted “V”‐shaped curve is also observed for N, S_v_‐ZIS/WS_2_. Moreover, the carrier concentration (*N*_d_) can be determined by the MS plot. *N*_d_ values of all samples were calculated through the following equation 2 [42]:

(2) $$N_{d}=\frac{dV}{d\left(1/c^{2}\right)}\;\frac{2}{e\varepsilon \varepsilon_{0}}=\frac{2}{e\varepsilon \varepsilon_{0}}\;\frac{1}{slope}$$

in which *e*, *ε*, *ε*_0_ represent the electronic charge, dielectric constant, and vacuum permittivity, respectively. The “slope” was obtained from the linear fitting data of the M‐S plot, specifically based on the linear slope of the MS plot at 5000 Hz (Figure S11). As evaluated in Figure 5e, *N*_d_ values N, S_v_‐ZIS/WS_2_ (5.54 × 10^21^ cm^−3^) are higher than those of ZIS (2.59 × 10^21^ cm^−3^) and N, S_v_‐ZIS (3.82 × 10^21^ cm^−3^) samples. This enhancement increases the availability of free electrons as charge carriers, which significantly improves the efficiency of photocatalytic reactions [43].

**FIGURE 6:**
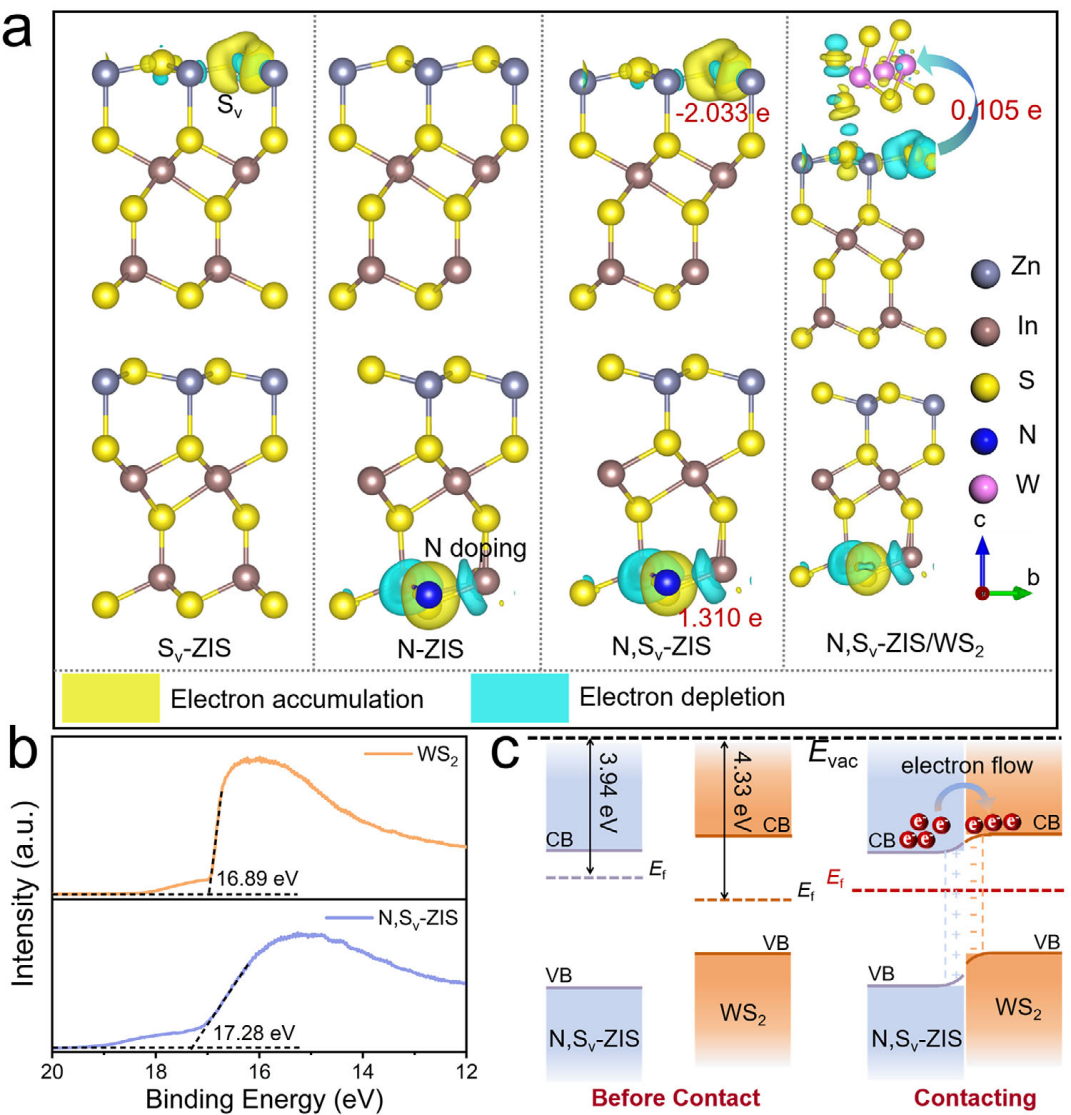
(a) Differential charge density diagram of S_v_‐ZIS, N‐ZIS, and N, S_v_‐ZIS samples. (b) UPS spectra of WS_2_ and N, S_v_‐ZIS samples. (c) Band structures of WS_2_ and N, S_v_‐ZIS before and after contact.

The innate character of the charge rearrangement and the possible direction of the BIEF in the N, S_v_‐ZIS can be elucidated by the three‐dimensional (3D) charge density difference. As shown in Figure 6a and Figure S12, electrons mainly accumulate in the N‐doped region and deplete in the S_v_‐engineered region, which can be displayed in cyan and yellow colors. This charge density discrepancy spontaneously establishes a BIEF‐like p‐n junction, extending from the S_v_ (n‐type) to the N‐doped (p‐type) sites. This intrinsic field configuration effectively facilitates the rapid separation and directional migration to S_v_ sites of photogenerated charge carriers in the catalytic reactions.

After compositing with WS_2_ (Figure 6a and Figure S12), the interfacial charge redistribution in N, S_v_‐ZIS/WS_2_ heterostructure manifests as electron depletion in N, S_v_‐ZIS domains and electron accumulation at WS_2_ regions, demonstrating interfacial coupling and intercomponent charge transfer. Bader charge analysis quantifies a net electron transfer of 0.105 e from ZIS to WS_2_, establishing a BIEF oriented from ZIS to WS_2_ across the heterojunction interface. To unambiguously determine interfacial charge transfer dynamics, we mapped the energy band alignment between N, S_v_‐ZIS, and WS_2_ through multi‐technique characterizations. UPS spectra with He I excitation (21.22 eV) revealed the cut‐off energies (*E*_cutoff_) of 17.28 and 16.89 eV for N, S_v_‐ZIS, and WS_2_, respectively (Figure 6b). The work functions (*Φ*), representing the energy difference from the *E*_f_ position to the vacuum level, were calculated as *Φ* = 21.22 eV‐ *E*_cutoff_, yielding values of 3.94 and 4.33 eV, which are close to the reported ZIS and 1T‐WS_2_ [11, 13, 21]. The energy band gaps (*E*_g_) derived from Tauc plots ${\left(\alpha hv\right)}^{\frac{1}{n}}=A\left(hv-E_{g}\right)$ were determined as 2.39 eV (N, S_v_‐ZIS) and 1.61 eV (WS_2_) (Figure S13). Additionally, as displayed in Figure S14, the valence band maxima (*E*_VBM_) values determined through the XPS valence spectra are about 1.16 and 0.26 eV (vs NHE). The conduction band minima (*E*_CBM_) were calculated by the formula *E*_CB_ = *E*_VB_—*E*_g_, respectively, as −1.23 eV (N, S_v_‐ZIS) and −1.35 eV (WS_2_). Based on the above comprehensive analysis, a type‐II staggered band alignment was constructed in N, S_v_‐ZIS/WS_2_ heterostructure after contacting (Figure 6c). Electrons transfer from ZIS (high *E*_f_) to WS_2_ (low *E*_f_) finally generates an interfacial BIEF, which is consistent with the simulated results.

### 2.6 Insights Into the Photocatalytic H_2_ Evolution Mechanism

**FIGURE 7:**
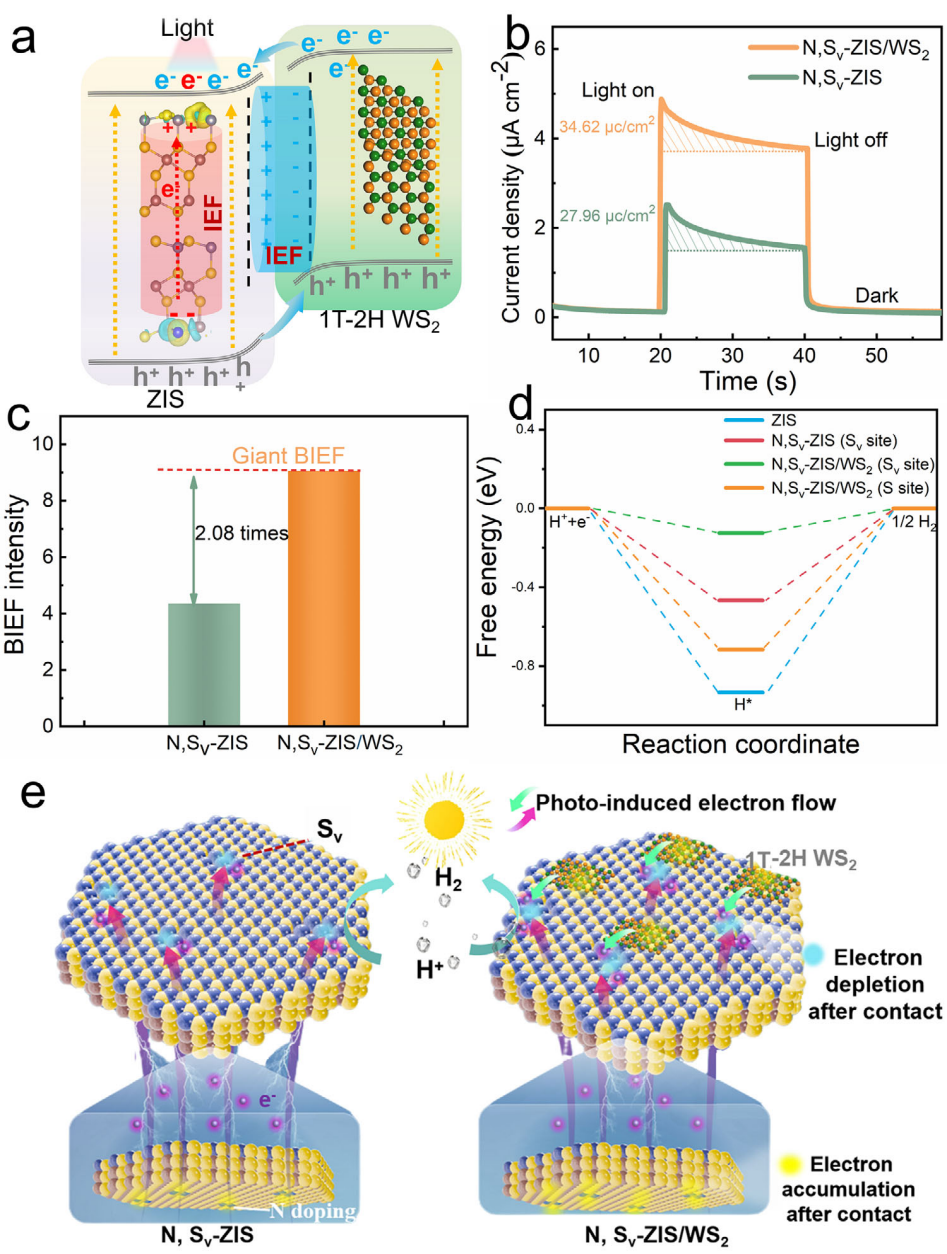
(a) Schematic diagram of photoinduced carrier transfer under the effect of internal and interfacial BIEFs. (b) The transient photocurrent density and (c) internal electric field intensity of N, S_v_‐ZIS, and N, S_v_‐ZIS/WS_2_ samples. (d) Hydrogen adsorption free energy of different samples. (e) Mechanism of photocatalytic H_2_ evolution over N, S_v_‐ZIS and N, S_v_‐ZIS/WS_2_ heterostructures.

As previously mentioned, in this ZIS‐based photocatalytic system, the introduction of S_v_ and N defects, as well as the incorporation of WS_2_ generates an internal p‐n type BIEF and an interfacial BIEF, respectively (Figure 7a). The directions of these two BIEFs are from S_v_ to the N‐doped sites in ZIS and from ZIS (S_v_ sites) to WS_2_, respectively, which are antiparallel. The stronger built‐in electric fields can effectively facilitate the separation of photogenerated carriers and guide the electron transfer to S_v_ sites. The intensity of their BIEF was calculated via the model (Equation 3) developed by Kanata‐Kito et al. [44, 45]:

(3) $$F_{s}={\left(-2\rho V_{S}/\varepsilon \varepsilon_{0}\right)}^{1/2}\;$$

where *F*_s_ is the internal electric field magnitude, *V*_s_ is the surface voltage, *ρ* is the surface charge density, ε is the low‐frequency dielectric constant, and *ε*_0_ is the permittivity of free space.

As shown in Figure 7b and Figure S15, the surface charge densities and surface voltages were determined by transient photocurrent density and open‐circuit potential measurements. The BIEF intensity of N, S_v_‐ZIS/WS_2_ is 2.08 times higher than that of N, S_v_‐ZIS (Figure 7c), indicating that the antiparallel dual‐BIEFs in N, S_v_‐ZIS/WS_2_ promote the formation of a faster charge carrier separation and transfer kinetics [44, 46], which has been verified by the results of photocurrent, EIS, TRPL, and photocatalytic performance. To further elucidate the surface catalytic contribution, the hydrogen adsorption free energy (ΔG_H_^*^) was calculated to evaluate the hydrogen evolution activity at different conditions (Figure 7d). Generally, the closer the ΔG_H_^*^ value is to zero, the higher the catalytic activity is [6]. The ΔG_H_^*^ of the S atom in the ZIS is −0.93 eV, suggesting the unfavorable hydrogen adsorption. Meanwhile, the ΔG_H_^*^ value of−0.47 eV acquired from the S_v_ site of N, S_v_‐ZIS indicates better hydrogen proton adsorption. Notably, the ΔG_H_^*^ value of the S_v_ site in the N, S_v_‐ZIS/WS_2_ sample is closer to zero. For the S atom in the N, S_v_‐ZIS/WS_2_, however, the ΔG_H_^*^ value is much lower at −0.72 eV. These results imply that S_v_ sites serve as active sites with optimized H adsorption/desorption, which is desirable for hydrogen evolution.

Based on the above characterization and analysis, we propose the photocatalytic hydrogen evolution mechanism of N, S_v_‐ZIS/WS_2_ (Figure 7e). Under light irradiation, the introduction of 1T‐2H WS_2_ enhances light absorption, promoting a significant increase in the generation of photogenerated carriers. Under the driving effect of interfacial BIEF, the photoinduced carriers in the ZIS‐WS_2_ interface are effectively separated, and the electrons originating from WS_2_ valence band are rapidly migrated to the conduction band of N, S_v_‐ZIS, and then directionally transferred to the N, S_v_‐ZIS surface to participate in the catalytic reaction. Meanwhile, the excellent electrical conductivity contributed by the metallic 1T phase of WS_2_ accelerates the process of carrier transfer. Notably, beyond the interfacial region, the photoinduced electrons that existed in the N, S_v_‐ZIS are also separated and transferred to the S_v_ sites in the ZIS surface under the influence of the internal p‐n type BIEF. As a result, the robust carrier separation and transfer dynamics are achieved, and the lifetime of these carriers is prolonged, attaining the significantly improved photocatalytic performance of N, S_v_‐ZIS/WS_2_.

## 3 Conclusion

In summary, an N, S_v_‐ZIS/WS_2_ composite photocatalyst was synthesized by a facile hydrothermal‐solvothermal method. S vacancies and N dopants were introduced into the ZIS lattice to obtain N, S_v_‐ZIS, which was further coupled with phase‐hybrid 1T‐2H WS_2_ to construct an interfacial heterostructure. This photocatalyst shows enhanced light absorption and improved charge separation, leading to a high photocatalytic performance with an H_2_ production rate of 44.97 mmol h^−1^ g^−1^ and the AQE of 24.54% at 400 nm. Experimental characterizations combined with DFT calculations confirm the formation of a p‐n homojunction within N, S_v_‐ZIS. After coupling with 1T‐2H WS_2_, a type‐II heterojunction is established at the N, S_v_‐ZIS/WS_2_ interface, giving rise to antiparallel internal and interfacial BIEFs. The dual‐BIEFs are stronger than the single BIEF in N, S_v_‐ZIS, enabling more efficient separation and directional transport of photogenerated charge carriers toward ZIS surface, where hydrogen evolution occurs. Overall, this work demonstrates that the integration of lattice doping, vacancy engineering, and hybrid‐phase heterointerface design effectively improves charge transfer and photocatalytic hydrogen evolution.

## 4 Experimental Section

### 4.1 Materials

Zinc acetate dihydrate (Zn(CH_3_COO)_2_·2H_2_O, 99.99% metal basis), Indium chloride (InCl_3_, 99.99% metal basis), thioacetamide (CH_3_CSNH_2_, TAA, ≥98%), tungsten chloride (WCl_6_, 99.9% metal basis), ascorbic acid (AA, >99%), N, N‐dimethylformamide (DMF, ≥99.9%) and Polyethylene glycol(HO(CH_2_CH_2_O)_n_H, PEG) were purchased from Aladdin Biochem. Tech. Co., LTD. All chemical reagents were obtained from commercial sources of analytical reagent (AR) grade without further purification.

### 4.2 Synthesis of ZIS and N, S_v_‐ZIS Samples

Pure ZIS was synthesized via a one‐step hydrothermal method. Specifically, 1 mmol InCl_3_, 0.5 mmol Zn(CH_3_COO)_2_·2H_2_O, and 4 mmol TAA were sequentially dissolved in a mixed solvent containing 10 mL deionized water and 5 mL PEG. After stirring the solution for 30 min, it was transferred into a 100 mL Teflon‐lined stainless steel autoclave. The autoclave was heated to 200°C and maintained at this temperature for 18 h. After natural cooling, the resulting product was collected by centrifugation, repeatedly washed with deionized water and ethanol, and then dried in an oven at 60°C for 10 h. The synthesis process of N, S_v_‐ZIS was basically consistent with that of pure ZIS, except that the 10 mL deionized water solvent in the reaction system was replaced with 10 mL DMF (i.e., the optimal introduction condition of N doping and sulfur vacancies determined by the previous optimization, corresponding to the optimization parameters of the pure DMF system in Figure S6), and all other reaction conditions remained unchanged.

### 4.3 Synthesis of N, S_v_‐ZIS/1T‐2H WS_2_ Composite Photocatalyst

To obtain the optimal composite ratio of ZIS and WS_2_ (which was systematically screened out in the previous comparative experiments and identified as the ratio corresponding to 64 mg WCl_6_), 64 mg of WCl_6_ and 75 mg of TAA were dissolved in a mixed solution of 10 mL DMF and 5 mL PEG. After complete dissolution under ultrasonication, the mixture was stirred at room temperature for 30 min. Subsequently, 1 mmol InCl_3_, 0.5 mmol Zn(CH_3_COO)_2_·2H_2_O and 4 mmol TAA were added sequentially—this dosage of InCl_3_, Zn(CH_3_COO)_2_·2H_2_O and TAA is consistent with the synthesis of pure ZIS and N, S_v_‐ZIS, ensuring that the composite ratio of ZIS to WS_2_ (regulated by 64 mg WCl_6_) remains the optimal value. The mixture was further dissolved via ultrasonication and stirred for another 30 min. The resulting solution was transferred into a 100 mL Teflon‐lined stainless steel autoclave and heated at 200°C for 18 h. After cooling to room temperature, the product was collected by centrifugation, repeatedly washed with deionized water and ethanol, and dried in a vacuum oven at 60°C for 10 h. The final product was designated as N, S_v_‐ZIS/1T‐2H WS_2_ (N, S_v_‐ZIS/WS_2_).

### 4.4 Characterization

The morphology and microstructure were characterized using a Sigma 300 scanning electron microscope (SEM) equipped with an Energy Dispersive Spectrometer (EDS), Talos F200X G2 transmission electron microscope (TEM). Crystal structure and phase information were analyzed by X‐ray diffraction (XRD) measurements (Smart Lab SE). The chemical composition and electronic states were investigated using an X‐ray photoelectron spectrometer (XPS, ESCALAB 250XI), with the C 1s peak at 284.8 eV serving as the reference for binding energy calibration. Ultraviolet photoelectron spectroscopy (UPS) measurements were conducted on an ESCALAB 250 Xi system to determine the cutoff energy values, with a photon emission energy of 21.22 eV through He I excitation.

The absorption properties were studied using a UV‐2550 spectrophotometer with barium sulfate as the reference standard. Raman spectroscopy analysis was carried out on a LabRAM HR Evolution system (Horiba) employing a 532 nm laser excitation source to probe molecular vibrations and chemical bonding. Electron spin resonance (ESR) spectra under dark and illuminated conditions were recorded on an EMXplus‐6/1 spectrometer operating at 9.054 GHz. Zeta potential measurements were performed using a Malvern Nano ZS90 potentiometric analyzer. Surface photovoltage and open‐circuit potential were evaluated through a CEL‐SPS1000 surface photovoltage system.

### 4.5 Photocatalysis Measurements

5 mg of photocatalyst was ultrasonically dispersed in 30 mL of 0.1 m ascorbic acid aqueous solution, forming a homogeneous suspension that was subsequently transferred into a sealed quartz reaction vessel. Before illumination, the reaction system underwent vacuum degassing for 30 min to eliminate dissolved oxygen and residual atmospheric gases. Photocatalytic reactions were initiated by irradiating the suspension with simulated solar light (AM 1.5G filter, 100 mW/cm^2^, spectral range 300–1100 nm) from a 300 W xenon lamp system. Temperature control at 8°C was maintained through a recirculating cooling system to minimize thermal effects. Evolved hydrogen gas was quantitatively analyzed at regular intervals using a GC 7900 gas chromatograph equipped with a thermal conductivity detector and nitrogen carrier gas, with calibration performed using standard hydrogen samples.

### 4.6 Photoelectrochemical and Electrochemical Measurements

To evaluate the photoelectrochemical performance of materials, a three‐electrode testing system using a Biologic SP‐200 electrochemical workstation was employed. The working electrode, the counter electrode, and the reference electrode were catalyst‐coated ITO conductive glass (2 cm × 3 cm), a platinum plate (1 cm^2^), and saturated Ag/AgCl (containing saturated potassium chloride electrolyte), respectively. The working electrode preparation involved: ultrasonically dispersing 10 mg of catalyst in 100 µL of anhydrous ethanol, uniformly coating the suspension onto the ITO substrate, followed by vacuum drying at 60°C for 1 h. The electrolyte was a 0.5 m Na_2_SO_4_ solution. The photocurrent response was tested under 0.4 V bias voltage and a 300 W Xenon lamp, and the light source automatically turned on/off every 20 s. MS plots were collected from −1.4 to 0.4 V under 1, 2, 3, and 5 kHz frequency and 0.005 V amplitude. EIS test was recorded in the frequency range from 0.01  to 10^5^ Hz with 0.005 V of the amplitude.

### 4.7 Calculations

All calculations were performed within the framework of density functional theory (DFT) by using the projector augmented wave method with the Perdew–Burke–Ernzerhof (PBE) exchange‐correlation functional [47, 48, 49]. The influence of vdW interactions is considered by using a modified version of vdW‐DF, referred to as ′optB86b‐vdW [50]. The projector augmented wave potentials were used with an energy cutoff of 600 eV. First, the bulk ZIS structure was fully optimized. A 12 ×  12 × 1 Monkhorst–Pack method k‐mesh was used for bulk geometry optimization. Energy convergence of 1.0 × 10^−4^ meV/atom was ensured during the self‐consistent field calculations. And the convergence criteria for the atomic forces were 0.01 eV/Å. The ZIS slab with S vacancy, N dopant, as well as WS_2_ heterojunction were respectively optimized. There exists a vacuum layer of more than 25 Å perpendicular to the surface plane. After geometry optimization, static calculation was performed. A 5 × 5 × 1 Monkhorst‐Pack method k‐mesh was used for slab geometry optimization and 9 × 9 × 1 for single point energy calculation.

### 4.8 Statistical Analysis

Statistical analysis of the data was conducted using *t*‐tests, and the results were presented as the mean ± standard deviation (SD). Independent experiments were replicated three times (n = 3). Statistical significance levels (^*^ *p* < 0.05, ^**^ *p* < 0.01, ^***^ *p* < 0.001) were defined through one‐way analysis of variance (ANOVA) using Origin software (version 2018).

## Conflicts of Interest

The authors declare no conflicts of interest.

## Supporting information




**Supporting File**: smll73203‐sup‐0001‐SuppMat.docx.

## Data Availability

The data that support the findings of this study are available from the corresponding author upon reasonable request.
