# Supplementary material for: Efficient Photocatalytic Hydrogen Evolution Enabled by Defect‐ and Interface‐Induced Dual Built‐in Electric Fields in a ZnIn2S4/1T‐2H WS2 Heterojunction
Source: Small. 2026 Mar 23;22(28):e14954. doi: 10.1002/smll.202514954 (PMC13181533; doi:10.1002/smll.202514954)
Supplement: Supplementary file 1 — Supporting File: smll73203‐sup‐0001‐SuppMat.docx. [file SMLL-22-e14954-s001.docx]

Supporting Information

**Efficient Photocatalytic Hydrogen Evolution Enabled by Defect- and Interface-Induced Dual Built-in Electric Fields in a ZnIn_2_S_4_/1T-2H WS_2_ Heterojunction**

*Ning Li*^#, *, 1^*, Jinyu Zhang* ^#, 1^*, Jiafeng Ma*^#, 1^*, Chaorui Xue*^1^*, Qing Chang*^1^*, Lei Liu*^1^*, Xiangqian Fan*^1^*, Caihong Hao*^1^*, Shaobin Wang*^2^*, Shengliang Hu*^*, 1^*, Wenjie Tian*^*, 2^

^1^School of Energy and Power Engineering & State Key Laboratory of Coal and CBM Co-Mining, North University of China, Taiyuan 030051, China

^2^School of Chemical Engineering, Adelaide University, Adelaide, SA 5005, Australia

Correspondence: Ning Li (lnlong2834@yeah.net); Shengliang Hu (hsliang@yeah.net);

Wenjie Tian (wenjie.tian@adelaide.edu.au)

^#^ Ning Li, Jinyu Zhang and Jiafeng Ma contributed equally to this work.


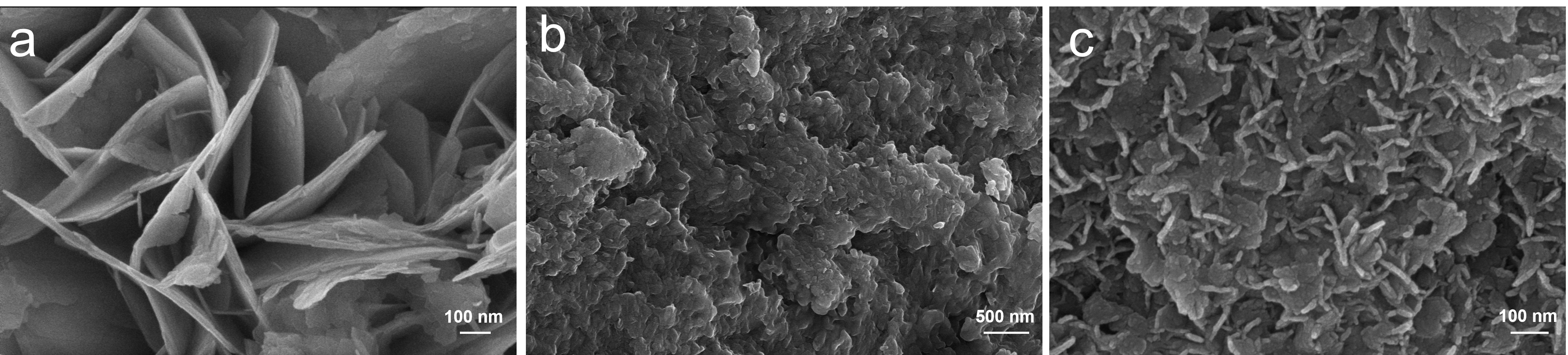


**FIGURE S1** ǀ SEM images of (a) ZIS, (b) N, S_v_-ZIS, and (c) N, S_v_-ZIS/WS_2_ samples.


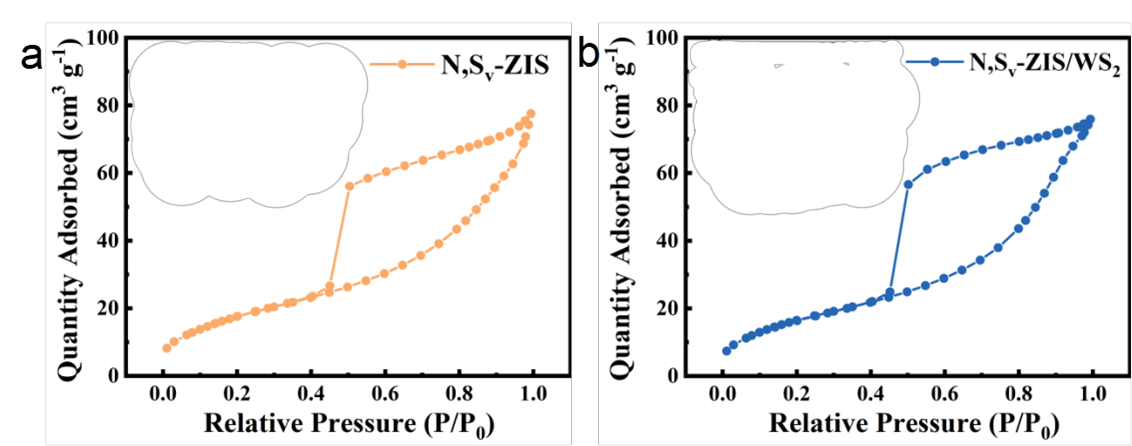


**FIGURE S2** ǀ N_2_ adsorption-desorption isotherms.


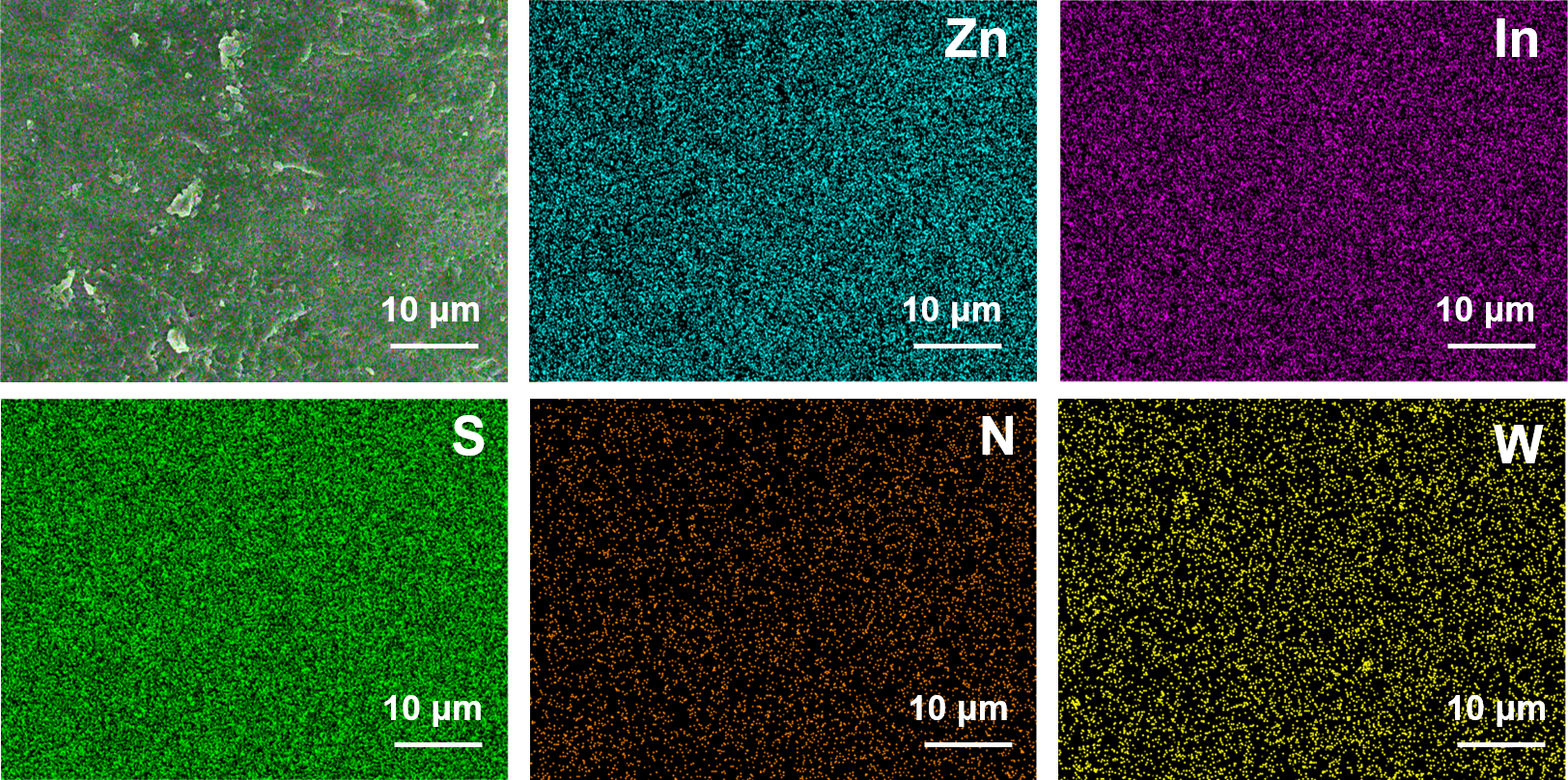


**FIGURE S3** ǀ SEM EDS mapping images of N, S_v_-ZIS/WS_2_.


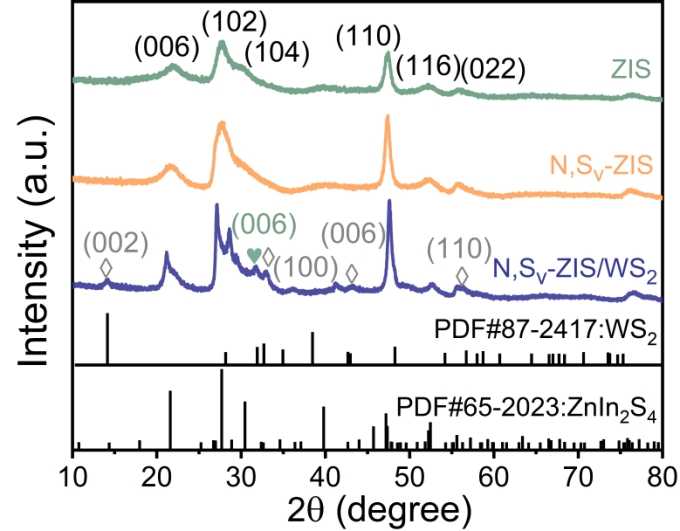


**FIGURE S4** ǀ XRD patterns of different samples.


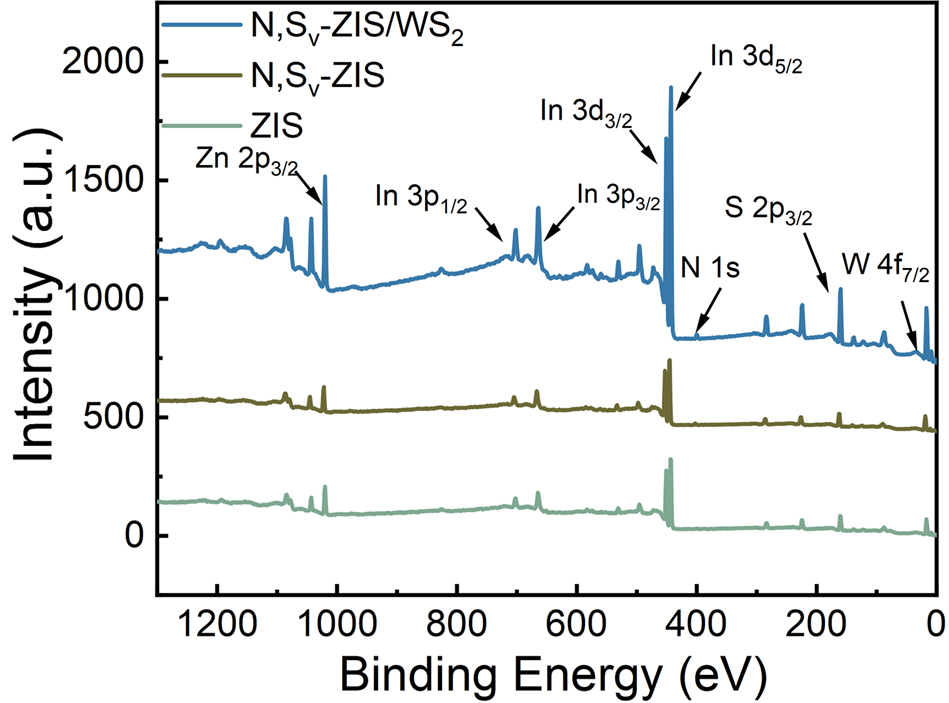


**FIGURE S5** ǀ XPS survey spectra.


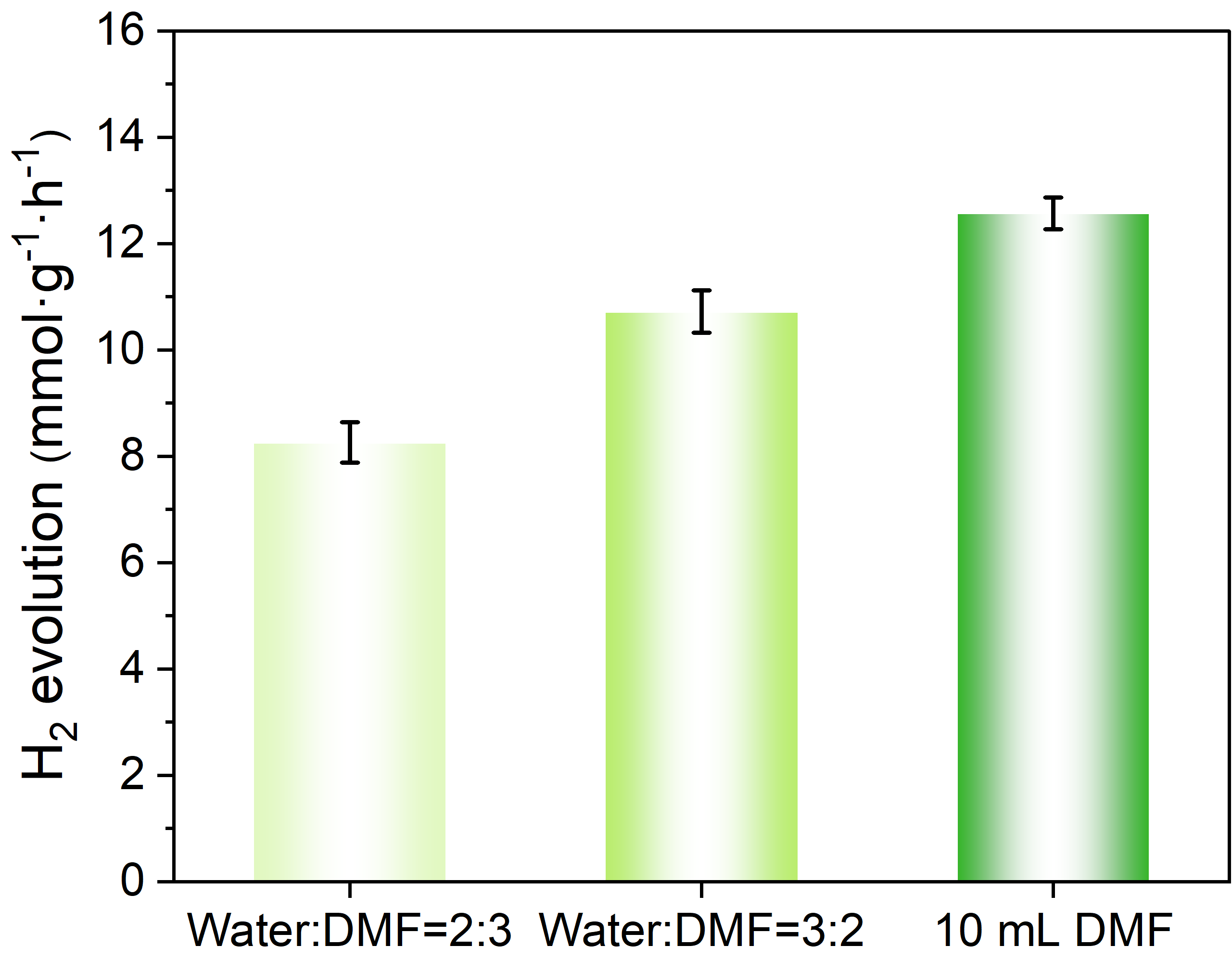


**FIGURE S6** ǀ Catalytic performance of N, S_v_-ZIS/WS_2_ catalysts with varied N and S_v_ level by adjust water/DMF ratio.


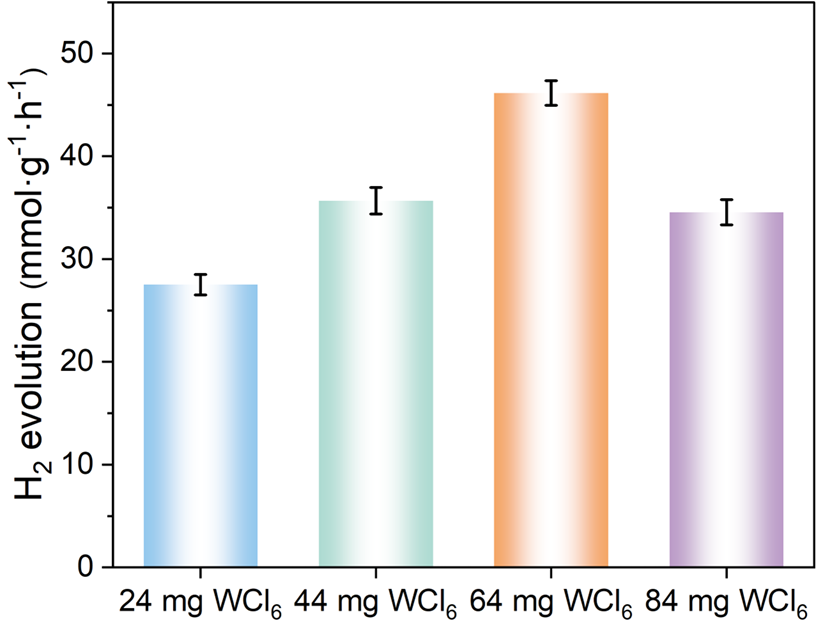


**FIGURE S7** ǀ Catalytic performance of N, S_v_-ZIS/WS_2_ with different ratios prepared with different amounts of WCl_6_.

The results show a clear N, S_v_-ZIS/WS_2_ ratio-dependent activity trend, with hydrogen evolution rates of 27.51, 35.68, 44.97, and 34.56 mmol·g^-1·^h^-1^ and the sample prepared with 64 mg WCl_6_ gives the best performance.


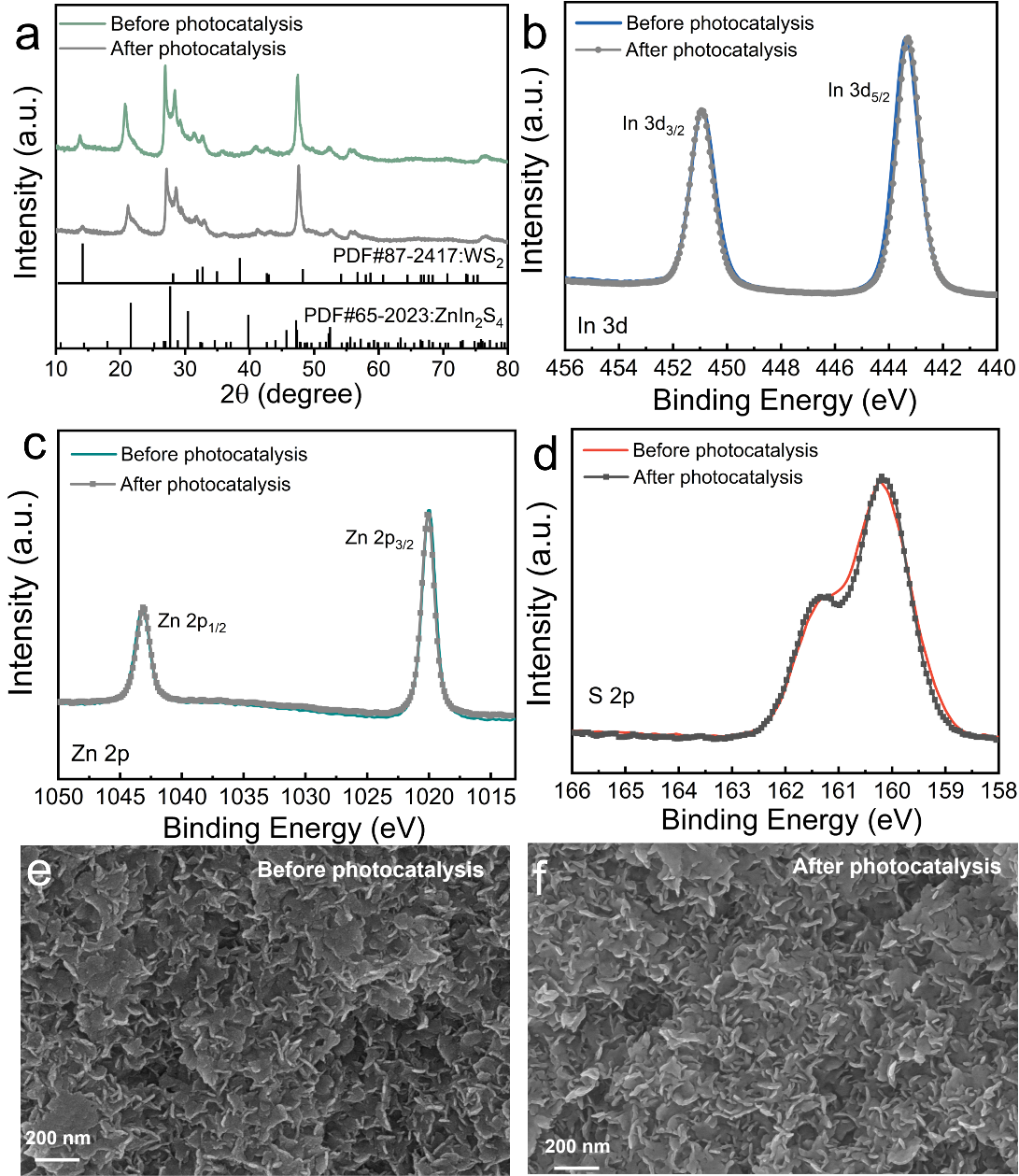


**FIGURE S8** ǀ **(**a) XRD patterns. (b-d) XPS spectra and (e, f) SEM images of N, S_v_-ZIS/WS_2_ before and after photocatalytic test.


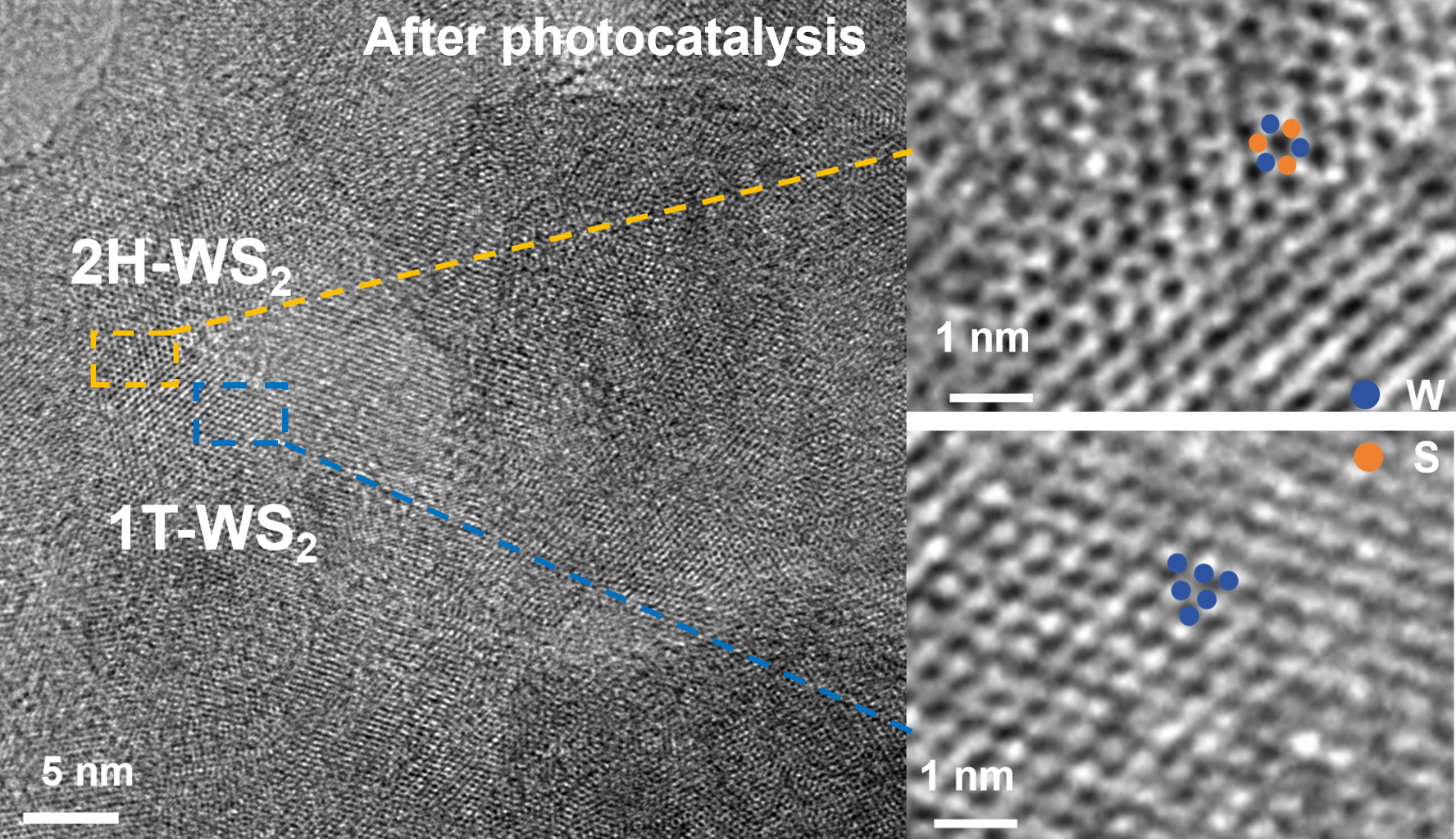


**FIGURE S9** ǀ HR-TEM images of N, S_v_-ZIS/WS_2_ after photocatalytic test.





**FIGURE S10** ǀ Wavelength-dependent AQE at the wavelength of 400, 420, 500 and 600 nm over N, S_v_-ZIS sample (Note: the error bars were based on three repeat experiments).


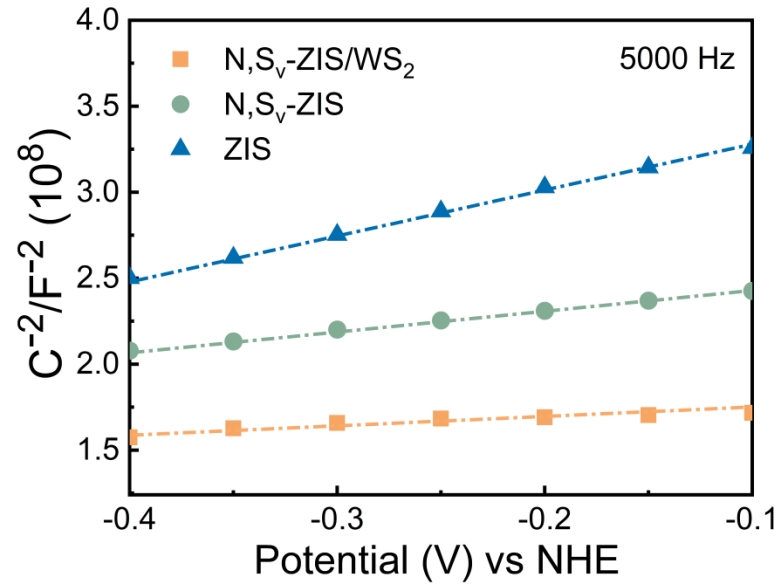


**FIGURE S11** ǀ Linearly fitted Mott-Schottky plot at 5000 Hz.


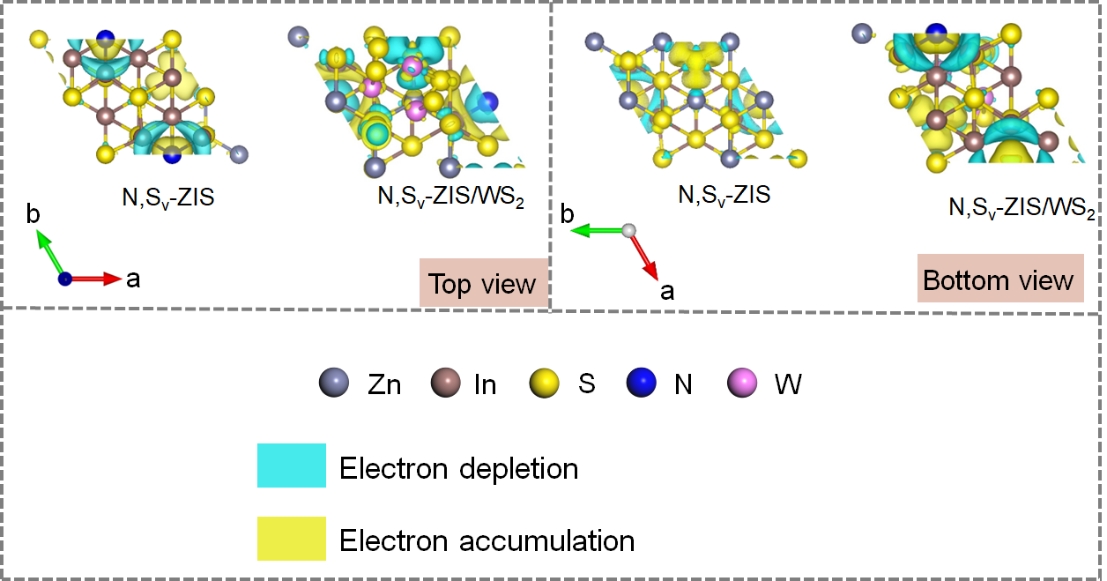


**FIGURE S12** ǀ Differential charge density from top and bottom views at the N, S_v_-ZIS/WS_2_ interface.


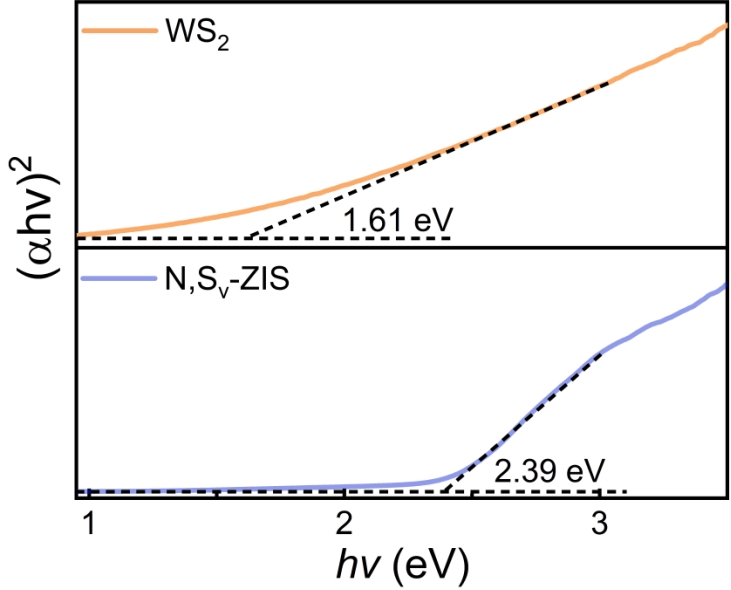


**FIGURE S13** ǀ Tauc plots of N, S_v_-ZIS and WS_2_.


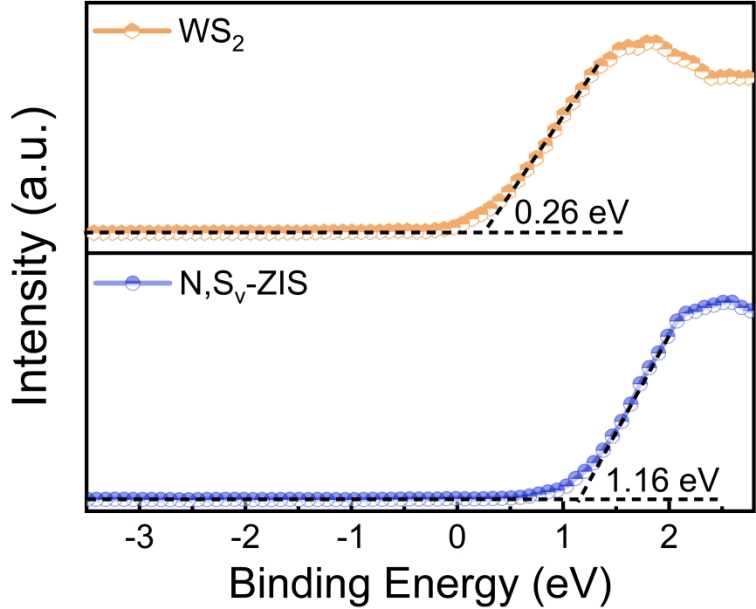


**FIGURE S14** ǀ Valence-band XPS spectra of N, S_v_-ZIS and WS_2_.


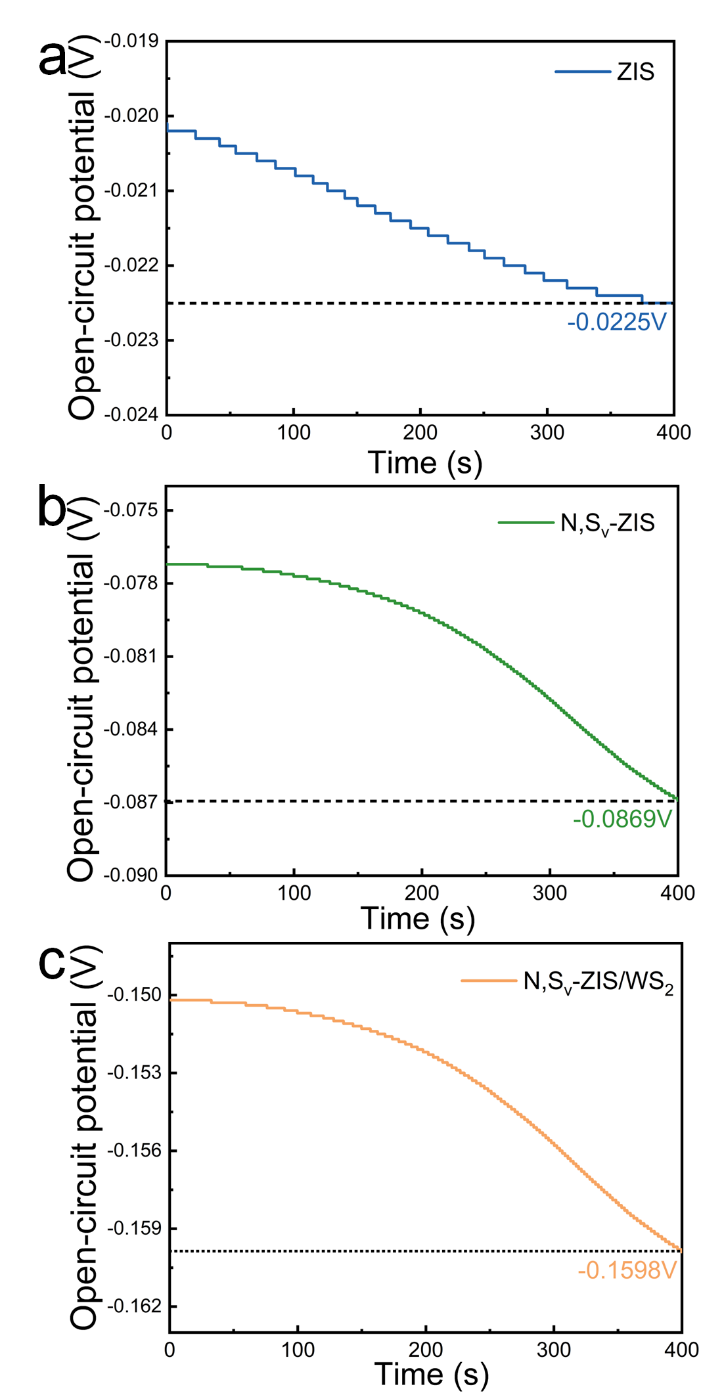


**FIGURE S15** ǀ The open-circuit potentials of ZIS, N, S_v_-ZIS and N, S_v_-ZIS/WS_2_ samples.

**Table S1** ǀ Specific surface area and pore structure parameters of different samples.

| Samples | Pore volume (cm^3^/g) | Average pore Size (nm) | SSA (m^2^/g) |
| --- | --- | --- | --- |
| N, S_v_-ZIS | 0.12 | 3.83 | 60.98 |
| N, S_v_-ZIS/WS_2_ | 0.12 | 4.17 | 68.35 |

**Table S2** ǀ Summary of the elemental contents of the samples.

| Sample | Zn (at.%) | In (at.%) | S (at.%) | N (at.%) | W (at.%) | Zn:In:S (molar ratio) |
| --- | --- | --- | --- | --- | --- | --- |
| N, S_v_-ZIS | 14.48 | 29.12 | 54.89 | 1.51 | - | 1:2.01:3.79 |
| ZIS | 14.26 | 28.67 | 57.07 | - | - | 1:2.01:4 |
| N, S_v_-ZIS/WS_2_ | 13.20 | 26.78 | 55.41 | 2.24 | 2.37 | - |

**Table S3** ǀ Detailed data for calculating AQE of different samples at different wavelengths.

| Wavelength | Photocatalytic H_2_ production | | Power |
| --- | --- | --- | --- |
|  | N, S_V_-ZIS/WS_2_ | N, S_V_-ZIS |  |
| 400 nm | 115.17±3.30 μmol | 34.59±0.99 μmol | 0.078 W |
| 420 nm | 102.04±1.60 μmol | 29.88±1 .26 μmol | 0.077 W |
| 500 nm | 22.45±0.81 μmol | 8.75±0.34 μmol | 0.051 W |
| 600 nm | 26.68±0.90 μmol | 9.52±0.40 μmol | 0.069 W |

Note: the error bars were based on three repeat experiments.

**The calculation of the apparent quantum efficiency (AQE)**

AQE was measured under similar conditions except that the incident light (400, 420, 500 and 600 nm) was monochromatic. The AQE of the MoS_2_/ZIS/GQDs sample were calculated by the following equation:^[S1, S2]^


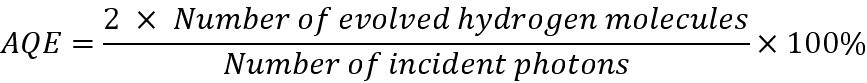


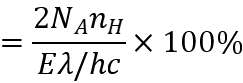


where *N*_A_ (6.02× 10^23^) is the Avogadro constant, *n*_H_ (mol/h) represents the H_2_ evolution rate, *E* (W) is the power of irradiation light, *λ* (nm) is the monochromatic wavelength (400, 420, 500 and 600 nm), *h* represents Planck constant (6.626 × 10^-34^ J·s), c (3 × 10^8^ m/s) is the speed of light.

N, S_V_-ZIS/WS_2_:

λ = 400 nm:


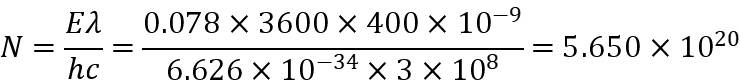


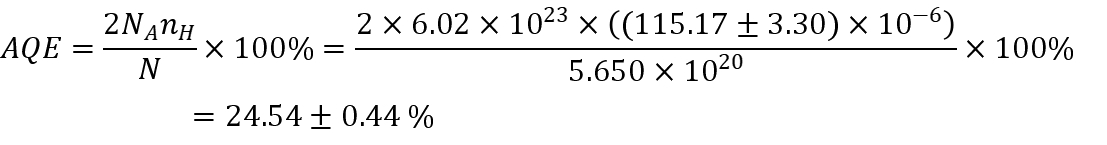


λ = 420 nm:


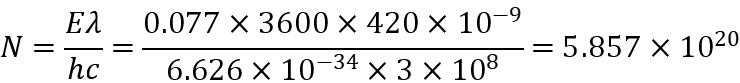


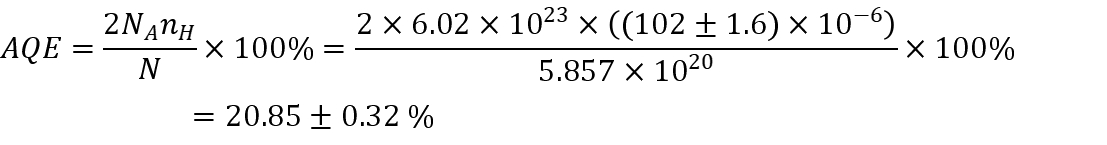


λ = 500 nm:


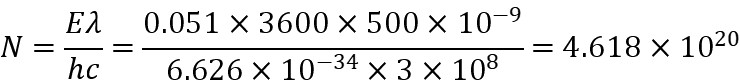


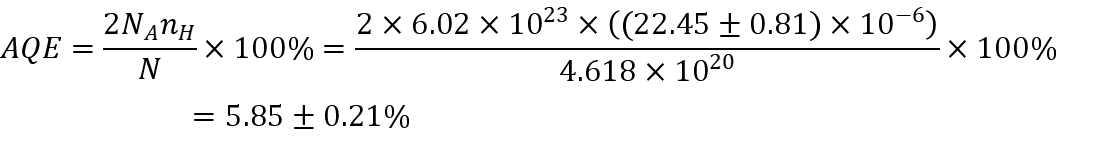


λ =600 nm:


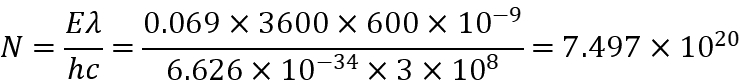


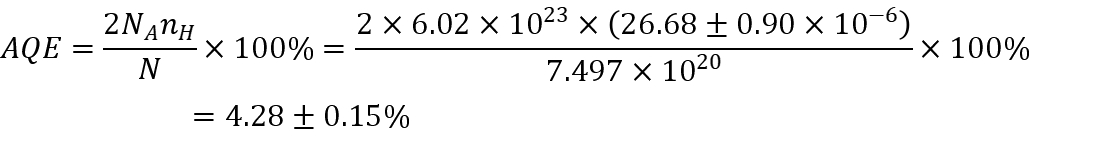


N, S_V_-ZIS:

λ = 400 nm:


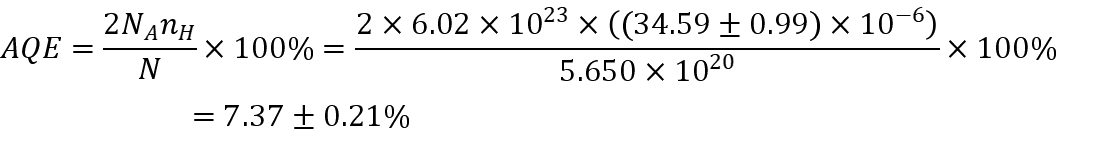


λ = 420 nm:


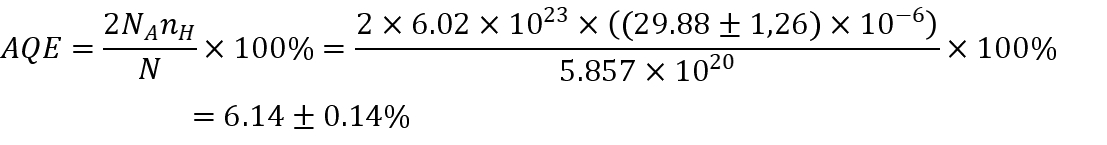


λ = 500 nm:


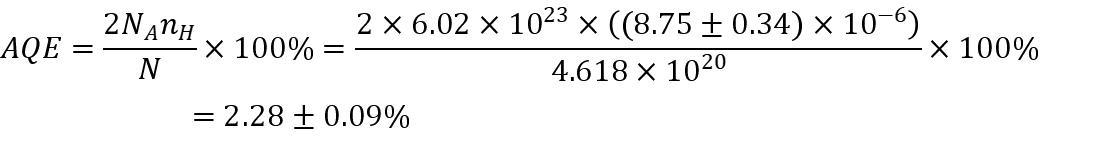


λ =600 nm:


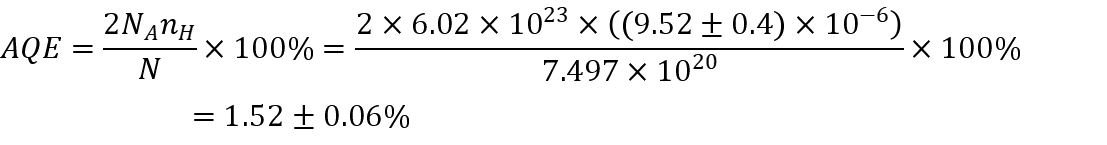


**Table S4-1** ǀ **Absorbance values of N, S_v_-ZIS/WS_2_ in Fig. 3d.**

| Wavelength (700-600 nm) | Absorbance (a.u.) | Wavelength (599-499 nm) | Absorbance (a.u.) | Wavelength (498-400 nm) | Absorbance (a.u.) |
| --- | --- | --- | --- | --- | --- |
| 700 | 0.20335 | 599 | 0.20639 | 498 | 0.2243 |
| 699 | 0.20343 | 598 | 0.20648 | 497 | 0.22531 |
| 698 | 0.20342 | 597 | 0.20657 | 496 | 0.22634 |
| 697 | 0.20343 | 596 | 0.20672 | 495 | 0.22745 |
| 696 | 0.20344 | 595 | 0.2068 | 494 | 0.22869 |
| 695 | 0.20351 | 594 | 0.20684 | 493 | 0.22999 |
| 694 | 0.20363 | 593 | 0.20691 | 492 | 0.23143 |
| 693 | 0.20375 | 592 | 0.20697 | 491 | 0.23293 |
| 692 | 0.20385 | 591 | 0.20702 | 490 | 0.23459 |
| 691 | 0.20395 | 590 | 0.20704 | 489 | 0.23643 |
| 690 | 0.20403 | 589 | 0.20712 | 488 | 0.23834 |
| 689 | 0.2042 | 588 | 0.20706 | 487 | 0.2405 |
| 688 | 0.20423 | 587 | 0.20712 | 486 | 0.24282 |
| 687 | 0.20429 | 586 | 0.2071 | 485 | 0.24534 |
| 686 | 0.20439 | 585 | 0.20714 | 484 | 0.24816 |
| 685 | 0.20447 | 584 | 0.20722 | 483 | 0.2511 |
| 684 | 0.20455 | 583 | 0.2072 | 482 | 0.2544 |
| 683 | 0.20453 | 582 | 0.20719 | 481 | 0.25789 |
| 682 | 0.20457 | 581 | 0.20723 | 480 | 0.26171 |
| 681 | 0.20459 | 580 | 0.20721 | 479 | 0.26586 |
| 680 | 0.20458 | 579 | 0.20721 | 478 | 0.27029 |
| 679 | 0.20454 | 578 | 0.20722 | 477 | 0.27519 |
| 678 | 0.20459 | 577 | 0.20728 | 476 | 0.28036 |
| 677 | 0.20454 | 576 | 0.20735 | 475 | 0.28584 |
| 676 | 0.20452 | 575 | 0.20744 | 474 | 0.29175 |
| 675 | 0.20446 | 574 | 0.20749 | 473 | 0.29791 |
| 674 | 0.20445 | 573 | 0.20763 | 472 | 0.30447 |
| 673 | 0.20445 | 572 | 0.20769 | 471 | 0.31145 |
| 672 | 0.20448 | 571 | 0.20769 | 470 | 0.31899 |
| 671 | 0.20443 | 570 | 0.20772 | 469 | 0.32692 |
| 670 | 0.20443 | 569 | 0.20774 | 468 | 0.33528 |
| 669 | 0.20445 | 568 | 0.2078 | 467 | 0.34411 |
| 668 | 0.20452 | 567 | 0.2078 | 466 | 0.35346 |
| 667 | 0.2045 | 566 | 0.20788 | 465 | 0.3632 |
| 666 | 0.20452 | 565 | 0.20798 | 464 | 0.37355 |
| 665 | 0.20455 | 564 | 0.20797 | 463 | 0.38449 |
| 664 | 0.2046 | 563 | 0.20798 | 462 | 0.39609 |
| 663 | 0.20462 | 562 | 0.20796 | 461 | 0.40824 |
| 662 | 0.20462 | 561 | 0.20809 | 460 | 0.42085 |
| 661 | 0.20468 | 560 | 0.2081 | 459 | 0.43412 |
| 660 | 0.20481 | 559 | 0.20811 | 458 | 0.44815 |
| 659 | 0.20489 | 558 | 0.20817 | 457 | 0.46257 |
| 658 | 0.20488 | 557 | 0.2082 | 456 | 0.47776 |
| 657 | 0.20492 | 556 | 0.20827 | 455 | 0.49343 |
| 656 | 0.20493 | 555 | 0.20827 | 454 | 0.50962 |
| 655 | 0.20499 | 554 | 0.20839 | 453 | 0.52631 |
| 654 | 0.20501 | 553 | 0.20837 | 452 | 0.54336 |
| 653 | 0.205 | 552 | 0.20839 | 451 | 0.56067 |
| 652 | 0.20503 | 551 | 0.20849 | 450 | 0.57835 |
| 651 | 0.20504 | 550 | 0.2086 | 449 | 0.59628 |
| 650 | 0.20501 | 549 | 0.20866 | 448 | 0.61423 |
| 649 | 0.20507 | 548 | 0.20871 | 447 | 0.63223 |
| 648 | 0.20506 | 547 | 0.20883 | 446 | 0.64997 |
| 647 | 0.20501 | 546 | 0.20888 | 445 | 0.66778 |
| 646 | 0.20499 | 545 | 0.20902 | 444 | 0.68534 |
| 645 | 0.205 | 544 | 0.20918 | 443 | 0.70293 |
| 644 | 0.20501 | 543 | 0.20939 | 442 | 0.72015 |
| 643 | 0.20503 | 542 | 0.20949 | 441 | 0.73677 |
| 642 | 0.20508 | 541 | 0.20956 | 440 | 0.75294 |
| 641 | 0.20503 | 540 | 0.2097 | 439 | 0.76869 |
| 640 | 0.20501 | 539 | 0.20984 | 438 | 0.78417 |
| 639 | 0.20501 | 538 | 0.20988 | 437 | 0.79921 |
| 638 | 0.20502 | 537 | 0.21 | 436 | 0.81389 |
| 637 | 0.20505 | 536 | 0.21007 | 435 | 0.82794 |
| 636 | 0.20502 | 535 | 0.21019 | 434 | 0.84136 |
| 635 | 0.20501 | 534 | 0.21034 | 433 | 0.85451 |
| 634 | 0.20501 | 533 | 0.21045 | 432 | 0.86698 |
| 633 | 0.20497 | 532 | 0.2105 | 431 | 0.87896 |
| 632 | 0.20496 | 531 | 0.21067 | 430 | 0.89105 |
| 631 | 0.20505 | 530 | 0.21074 | 429 | 0.90283 |
| 630 | 0.20512 | 529 | 0.21093 | 428 | 0.91441 |
| 629 | 0.20519 | 528 | 0.21117 | 427 | 0.92556 |
| 628 | 0.20532 | 527 | 0.21132 | 426 | 0.93669 |
| 627 | 0.20539 | 526 | 0.21158 | 425 | 0.94735 |
| 626 | 0.20551 | 525 | 0.21174 | 424 | 0.95742 |
| 625 | 0.2056 | 524 | 0.21204 | 423 | 0.96732 |
| 624 | 0.20569 | 523 | 0.21226 | 422 | 0.97689 |
| 623 | 0.20578 | 522 | 0.21247 | 421 | 0.98675 |
| 622 | 0.20591 | 521 | 0.2128 | 420 | 0.99573 |
| 621 | 0.20591 | 520 | 0.21299 | 419 | 1.00447 |
| 620 | 0.206 | 519 | 0.21331 | 418 | 1.01305 |
| 619 | 0.20603 | 518 | 0.21363 | 417 | 1.02125 |
| 618 | 0.20607 | 517 | 0.21388 | 416 | 1.02942 |
| 617 | 0.20608 | 516 | 0.21429 | 415 | 1.03741 |
| 616 | 0.20608 | 515 | 0.21461 | 414 | 1.0456 |
| 615 | 0.20616 | 514 | 0.21495 | 413 | 1.0529 |
| 614 | 0.20624 | 513 | 0.21523 | 412 | 1.06104 |
| 613 | 0.20624 | 512 | 0.21562 | 411 | 1.06797 |
| 612 | 0.20616 | 511 | 0.21595 | 410 | 1.07506 |
| 611 | 0.20609 | 510 | 0.21634 | 409 | 1.08192 |
| 610 | 0.20605 | 509 | 0.21677 | 408 | 1.08823 |
| 609 | 0.20599 | 508 | 0.21723 | 407 | 1.09411 |
| 608 | 0.20596 | 507 | 0.21769 | 406 | 1.09918 |
| 607 | 0.20603 | 506 | 0.21822 | 405 | 1.10463 |
| 606 | 0.20604 | 505 | 0.21877 | 404 | 1.10961 |
| 605 | 0.20605 | 504 | 0.21934 | 403 | 1.11459 |
| 604 | 0.20607 | 503 | 0.22008 | 402 | 1.1187 |
| 603 | 0.20618 | 502 | 0.22088 | 401 | 1.12355 |
| 602 | 0.20621 | 501 | 0.2216 | 400 | 1.12726 |
| 601 | 0.20632 | 500 | 0.22245 |  |  |
| 600 | 0.20638 | 499 | 0.22334 |  |  |

**Table S4-2** ǀ **Absorbance values of N, S_v_-ZIS in Figure S10.**

| Wavelength (700-600 nm) | Absorbance (a.u.) | Wavelength (599-499 nm) | Absorbance (a.u.) | Wavelength (498-400 nm) | Absorbance (a.u.) |
| --- | --- | --- | --- | --- | --- |
| 700 | 0.04465 | 599 | 0.05074 | 498 | 0.15764 |
| 699 | 0.04469 | 598 | 0.05092 | 497 | 0.16129 |
| 698 | 0.04469 | 597 | 0.05107 | 496 | 0.16513 |
| 697 | 0.04469 | 596 | 0.05124 | 495 | 0.16903 |
| 696 | 0.04469 | 595 | 0.05148 | 494 | 0.17312 |
| 695 | 0.04478 | 594 | 0.05162 | 493 | 0.17734 |
| 694 | 0.04489 | 593 | 0.05183 | 492 | 0.18172 |
| 693 | 0.04495 | 592 | 0.05204 | 491 | 0.18619 |
| 692 | 0.04501 | 591 | 0.0522 | 490 | 0.19078 |
| 691 | 0.04499 | 590 | 0.05227 | 489 | 0.19556 |
| 690 | 0.04495 | 589 | 0.05236 | 488 | 0.20042 |
| 689 | 0.04497 | 588 | 0.05247 | 487 | 0.2055 |
| 688 | 0.04494 | 587 | 0.05263 | 486 | 0.21076 |
| 687 | 0.04491 | 586 | 0.05274 | 485 | 0.21607 |
| 686 | 0.04497 | 585 | 0.05285 | 484 | 0.22164 |
| 685 | 0.04511 | 584 | 0.05309 | 483 | 0.22734 |
| 684 | 0.04513 | 583 | 0.05324 | 481 | 0.23913 |
| 683 | 0.04514 | 582 | 0.05337 | 480 | 0.24524 |
| 682 | 0.04528 | 581 | 0.05355 | 479 | 0.25159 |
| 681 | 0.04535 | 580 | 0.05366 | 478 | 0.25802 |
| 680 | 0.04537 | 579 | 0.05387 | 477 | 0.26475 |
| 679 | 0.04541 | 578 | 0.05408 | 476 | 0.27156 |
| 678 | 0.04554 | 577 | 0.05436 | 475 | 0.27848 |
| 677 | 0.04566 | 576 | 0.0546 | 474 | 0.28551 |
| 676 | 0.04573 | 575 | 0.05482 | 473 | 0.29272 |
| 675 | 0.04579 | 574 | 0.05509 | 472 | 0.30004 |
| 674 | 0.04596 | 573 | 0.05537 | 471 | 0.30757 |
| 673 | 0.04609 | 572 | 0.05567 | 470 | 0.31517 |
| 672 | 0.04618 | 571 | 0.05598 | 469 | 0.32285 |
| 671 | 0.04628 | 570 | 0.05624 | 468 | 0.33078 |
| 670 | 0.04637 | 569 | 0.05651 | 467 | 0.33894 |
| 669 | 0.04642 | 568 | 0.05676 | 466 | 0.34716 |
| 668 | 0.04645 | 567 | 0.05706 | 465 | 0.35561 |
| 667 | 0.04645 | 566 | 0.05734 | 464 | 0.36424 |
| 666 | 0.04651 | 565 | 0.05763 | 463 | 0.37318 |
| 665 | 0.0465 | 564 | 0.05798 | 462 | 0.38229 |
| 664 | 0.04646 | 563 | 0.05833 | 461 | 0.3916 |
| 663 | 0.04642 | 562 | 0.05857 | 460 | 0.40097 |
| 662 | 0.0464 | 561 | 0.05901 | 459 | 0.4105 |
| 661 | 0.04631 | 560 | 0.05937 | 458 | 0.42021 |
| 660 | 0.04629 | 559 | 0.05976 | 457 | 0.43008 |
| 659 | 0.0463 | 558 | 0.06016 | 456 | 0.43996 |
| 658 | 0.0462 | 557 | 0.06057 | 455 | 0.45009 |
| 657 | 0.04621 | 556 | 0.06096 | 454 | 0.46013 |
| 656 | 0.0462 | 555 | 0.06138 | 453 | 0.47026 |
| 655 | 0.04628 | 554 | 0.06182 | 452 | 0.48039 |
| 654 | 0.04632 | 553 | 0.06234 | 451 | 0.49044 |
| 653 | 0.04645 | 552 | 0.06283 | 450 | 0.50063 |
| 652 | 0.04653 | 551 | 0.06339 | 449 | 0.51074 |
| 651 | 0.04664 | 550 | 0.06395 | 448 | 0.5209 |
| 650 | 0.04668 | 549 | 0.06453 | 447 | 0.53104 |
| 649 | 0.04674 | 548 | 0.06511 | 446 | 0.54072 |
| 648 | 0.04673 | 547 | 0.06577 | 445 | 0.55011 |
| 647 | 0.04682 | 546 | 0.06651 | 444 | 0.55974 |
| 646 | 0.04686 | 545 | 0.06723 | 443 | 0.56889 |
| 645 | 0.04689 | 544 | 0.06805 | 442 | 0.57802 |
| 644 | 0.04697 | 543 | 0.06885 | 441 | 0.58678 |
| 643 | 0.04708 | 542 | 0.06971 | 440 | 0.59506 |
| 642 | 0.04717 | 541 | 0.07057 | 439 | 0.603 |
| 641 | 0.04714 | 540 | 0.07143 | 438 | 0.61061 |
| 640 | 0.04721 | 539 | 0.0723 | 437 | 0.61794 |
| 639 | 0.04718 | 538 | 0.07317 | 436 | 0.6251 |
| 638 | 0.04721 | 537 | 0.07415 | 435 | 0.63219 |
| 637 | 0.04729 | 536 | 0.07512 | 434 | 0.63891 |
| 636 | 0.0473 | 535 | 0.07625 | 433 | 0.6453 |
| 635 | 0.0473 | 534 | 0.07736 | 432 | 0.65158 |
| 634 | 0.0473 | 533 | 0.07849 | 431 | 0.65743 |
| 633 | 0.04729 | 532 | 0.07972 | 430 | 0.66325 |
| 632 | 0.04738 | 531 | 0.08102 | 429 | 0.66879 |
| 631 | 0.04739 | 530 | 0.08229 | 428 | 0.67396 |
| 630 | 0.04739 | 529 | 0.08365 | 427 | 0.67897 |
| 629 | 0.04752 | 528 | 0.0851 | 426 | 0.68402 |
| 628 | 0.04763 | 527 | 0.08655 | 425 | 0.68888 |
| 627 | 0.04767 | 526 | 0.08813 | 424 | 0.69347 |
| 626 | 0.04787 | 525 | 0.08973 | 423 | 0.69824 |
| 625 | 0.04802 | 524 | 0.09138 | 422 | 0.703 |
| 624 | 0.0482 | 523 | 0.09307 | 421 | 0.70762 |
| 623 | 0.04828 | 522 | 0.09482 | 420 | 0.71212 |
| 622 | 0.0484 | 521 | 0.09666 | 419 | 0.71645 |
| 621 | 0.04847 | 520 | 0.09849 | 418 | 0.72062 |
| 620 | 0.04863 | 519 | 0.10054 | 417 | 0.72479 |
| 619 | 0.04871 | 518 | 0.10258 | 416 | 0.72901 |
| 618 | 0.04881 | 517 | 0.10467 | 415 | 0.73312 |
| 617 | 0.04886 | 516 | 0.10678 | 414 | 0.73683 |
| 616 | 0.04891 | 515 | 0.10899 | 413 | 0.74014 |
| 615 | 0.04905 | 514 | 0.11123 | 412 | 0.74303 |
| 614 | 0.0491 | 513 | 0.11352 | 411 | 0.74558 |
| 613 | 0.04924 | 512 | 0.11592 | 410 | 0.74829 |
| 612 | 0.04923 | 511 | 0.11832 | 409 | 0.75084 |
| 611 | 0.04935 | 510 | 0.12081 | 408 | 0.75317 |
| 610 | 0.04942 | 509 | 0.12338 | 407 | 0.75564 |
| 609 | 0.04948 | 508 | 0.12597 | 406 | 0.75791 |
| 608 | 0.04955 | 507 | 0.12873 | 405 | 0.75987 |
| 607 | 0.04961 | 506 | 0.13153 | 404 | 0.7612 |
| 606 | 0.04969 | 505 | 0.13444 | 403 | 0.76282 |
| 605 | 0.04988 | 504 | 0.13746 | 402 | 0.76499 |
| 604 | 0.04994 | 503 | 0.14061 | 401 | 0.76646 |
| 603 | 0.05011 | 502 | 0.14388 | 400 | 0.76784 |
| 602 | 0.05028 | 501 | 0.14717 |  |  |
| 601 | 0.05044 | 500 | 0.15061 |  |  |
| 600 | 0.05062 | 499 | 0.15413 |  |  |

**Table S5** ǀ **Photocatalytic hydrogen evolution performance and relevant experimental parameters of ZIS-based catalysts with sulfur vacancies (data used in Figure 3e).**

| Catalyst | Light  source | Sacrificial agent | Catalyst loading | Reactor volume | H_2_ production  (mmol/g/h) | Ref. in the main text |
| --- | --- | --- | --- | --- | --- | --- |
| N,S_v_-ZIS | 300 W Xe lamp(AM 1.5G filter, 100 mW/cm^2^, spectral range 300-1100 nm) | 0.1 M AA | 5 mg | 100 mL | 12.57 | This work |
| N, S_v_-ZIS/WS_2_ |  |  |  |  | 44.97 |  |
| Ni-ZIS | 300 W Xe lamp with a 400 nm cut-off filter | 10 vol% TEOA | 15 mg | 50 mL | 18.19 | [8] |
| PC@MoS_2_/ZIS | 300 W Xe lamp with an AM 1.5 filter served as the simulated solar light source (100 mW cm^−2^) | 0.1 M AA | 5 mg | 30 mL | 18.79 | [11] |
| S_v_-ZIS | 300 W Xe lamp with a 420 nm cut-off filter | 0.1 M AA | 50 mg | 100 mL | 4.77 | [13] |
| N-doped ZIS | 350 W Xe lamp with an optical filter (λ ≥ 400nm) | 0.35 M Na_2_S + 0.35 M Na_2_SO_3_ | 30 mg | 60 mL | 1.58 | [14] |
| N-doped ZIS | 300 W Xe lamp under 470 nm light | 10 vol% TEOA | 20 mg | 100 mL | 11.09 | [15] |
| MoS_2_/S_v_-ZIS | 300 W Xe lamp equipped with a filter (λ > 420 nm) | 6.67 g‧L^-1^α-cellulose | 100 mg | 150 mL | 1.57 | [16] |
| ZIS-Mxene | 300 W Xe lamp with a filter (λ > 420 nm). | 10 vol% TEOA | 10 mg | 100 mL | 14.82 | [18] |
| 1T-WS_2_/ZIS | 300 W Xe lamp (AM 1.5), | 20 vol% AA | 10 mg | 50 mL | 30.90 | [22] |
| S_v_-ZIS | 300 W Xe lampequipped with a 420 nm cut-off filter | 0.1 M AA | 5 mg | 100 mL | 3.73 | [23] |
| S_v_-ZIS/In_2_Se_3_ | 300 W Xe lampequipped with a 420 nm cut-off filter | 0.1 M AA | 5 mg | 100 mL | 36.53 | [23] |
| S_v_-ZIS/TpPa-1 | 300 W Xe lamp (λ>420 nm) | 0.05M L-Ascorbic Acid | 25 mg | 50 mL | 2.75 | [26] |
| MoS_2_/ZIS/GQDs | 300 W Xe lamp (100 mW/cm^2^, AM 1.5G, 300-1100 nm) | 0.1 M AA | 5 mg | 100 mL | 21.63 | [32] |
| ZIS/WS_2_ | 150 W Xe lamp (Newport Model 6256, and light source 10 cm from the vessel) | 0.35 M Na_2_S + 0.25 M Na_2_SO_3_ | 50 mg | 100 mL | 0.29 | [33] |
| WS_2_/Zn_3_In_2_S_6_ | 300 W Xe lamp | 0.35 M Na_2_S + 0.35 M NaH_2_PO_2_ | 10 mg | 50 mL | 30.21 | [34] |
| Zn_v_, S_v_-ZIS | 300 W Xe lamp (λ > 420 nm) | 10 M BZA | 50 mg | 100 mL | 4.65 | [35] |
| S_v_-ZIS | 300 W Xe lamp (PLS-SXE300UV) equipped with a cut-off filter (λ> 420 nm) | 15 vol% TEOA | 5 mg | 10 mL | 2.40 | [36] |
| V, S_v_-ZIS | 300 W Xe lamp (model CEL-HXF300-T3) fitted with a 420 nm cut-off filter | 20 vol% TEOA | 10 mg | 10 mL | 4.94 | [37] |

**References**

# [S1] X. Wang, X. Wang, H. Yang, A. Meng, Z. Li, L. Yang, L. Wang, S. Li, G. Li, J. Huang, “Interfacial engineering improved internal electric field contributing to direct Z-scheme-dominated mechanism over CdSe/SL-ZnIn_2_S_4_/MoSe_2_ heterojunction for efficient photocatalytic hydrogen evolution,” *Chemical Engineering Journal* 431 (2022): 134000.

# [S2] Y. Peng, M. Geng, J. Yu, Y. Zhang, F. Tian, Y. Guo, D. Zhang, X. Yang, Z. Li, Z. Li, S. Zhang, “Vacancy-induced 2H@1T MoS_2_ phase-incorporation on ZnIn_2_S_4_ for boosting photocatalytic hydrogen evolution,” *Applied Catalysis B: Environmental* 298 (2021): 120570.
